# Chitons (Mollusca, Polyplacophora) from Alacranes Reef, Yucatan, Mexico

**DOI:** 10.3897/zookeys.665.10476

**Published:** 2017-04-03

**Authors:** Adriana Reyes-Gómez, Deneb Ortigosa, Nuno Simões

**Affiliations:** 1 Departamento de Ecología, Centro Universitario de Ciencias Biológicas y Agropecuarias, Universidad de Guadalajara, Carretera a Nogales km. 15.5, Las Agujas Nextipac C.P. 45110, Zapopan, Jalisco, México; 2 UMDI-Sisal, Facultad de Ciencias, Universidad Nacional Autónoma de México. Apartado postal 70-153, C.P. 04510, Ciudad de México, México; 3 Departamento de Biología, Facultad de Ciencias del Mar y Ambientales, Universidad de Cádiz, Polígono del Rio San Pedro s/n, Apartado 40, C.P. 11510, Puerto Real, Cádiz, Spain

**Keywords:** Biodiversity, marine reserves, coral reef, mollusks, Campeche Bank, Gulf of Mexico

## Abstract

This study represents the first comprehensive chiton study from Alacranes Reef, the largest reef system in the Gulf of Mexico. Nine chiton species were found in seven localities within the area, in the intertidal and subtidal to 12 m depth. SEM examination of *C. janeirensis*, *A. hemphilli*, *T. schrammi* and *C. floridanus*, showed variations in the sculpture and radular teeth morphology when compared to specimens of the same species from Florida Keys, Bahamas and Puerto Rico. The distribution ranges of *T. schrammi*, *L. liozonis* and *S. floridana* are extended into the south-western area of the Gulf of Mexico. Altogether, combining previous literature and the present survey, reports eleven chiton species which have now been recorded within the Alacranes reef area.

## Introduction

The Gulf of Mexico has diverse coastal geomorphology, climate and hydrology processes (Wiley et al. 1982). The Alacranes (or Scorpion) Reef National Park (the acronym, PNAA, refers to the Spanish name: Parque Nacional Arrecife Alacranes) is the largest coral reef in the Gulf of Mexico, with a unique atoll shape and is recognized as a Marine Protected Area. It is located 135 km north of Puerto Progreso, Yucatan, and it is considered the best-known Mexican reef, for its accumulated geological and paleontological data sets (e.g. Fosberg 1961, Chavez et al. 1985). Malacological studies at the PNAA have been mainly focused on bivalve and gastropod species (Rice and Kornicker 1962, 1965, Ekdale 1974, Boudreaux 1987, González et al. 1991, Hicks et al. 2001, Sanvicente-Añorve et al. 2012a, 2012b, Ortigosa et al. 2015). Chitons have been included only in species listings, based on their observed occurrences in areas near to the PNAA, especially in Mexican states of Yucatan and Quintana Roo (Ekdale 1974, Vokes and Vokes 1983, Watters 1981, 1990, Bullock 1985, 1988, Lyons 1985, 1988, Hicks et al. 2001, Reyes-Gómez and Salcedo-Vargas 2002, Lyons and Moretzsohn 2009). For the Mexican Caribbean, 19 species are known whereas only six have been recorded from particular localities within the PNAA. Here, as in much of the world, chiton species tallies have been based so far on only morphological comparisons, mostly from details of the shell plates (valves) as supplemented by girdle and radular tooth features (Irisarri et al. 2014). In this study, we described and figured these morphological characters and update the chiton checklist for the Alacranes Reef.

## Materials and methods

**Figure 1.**
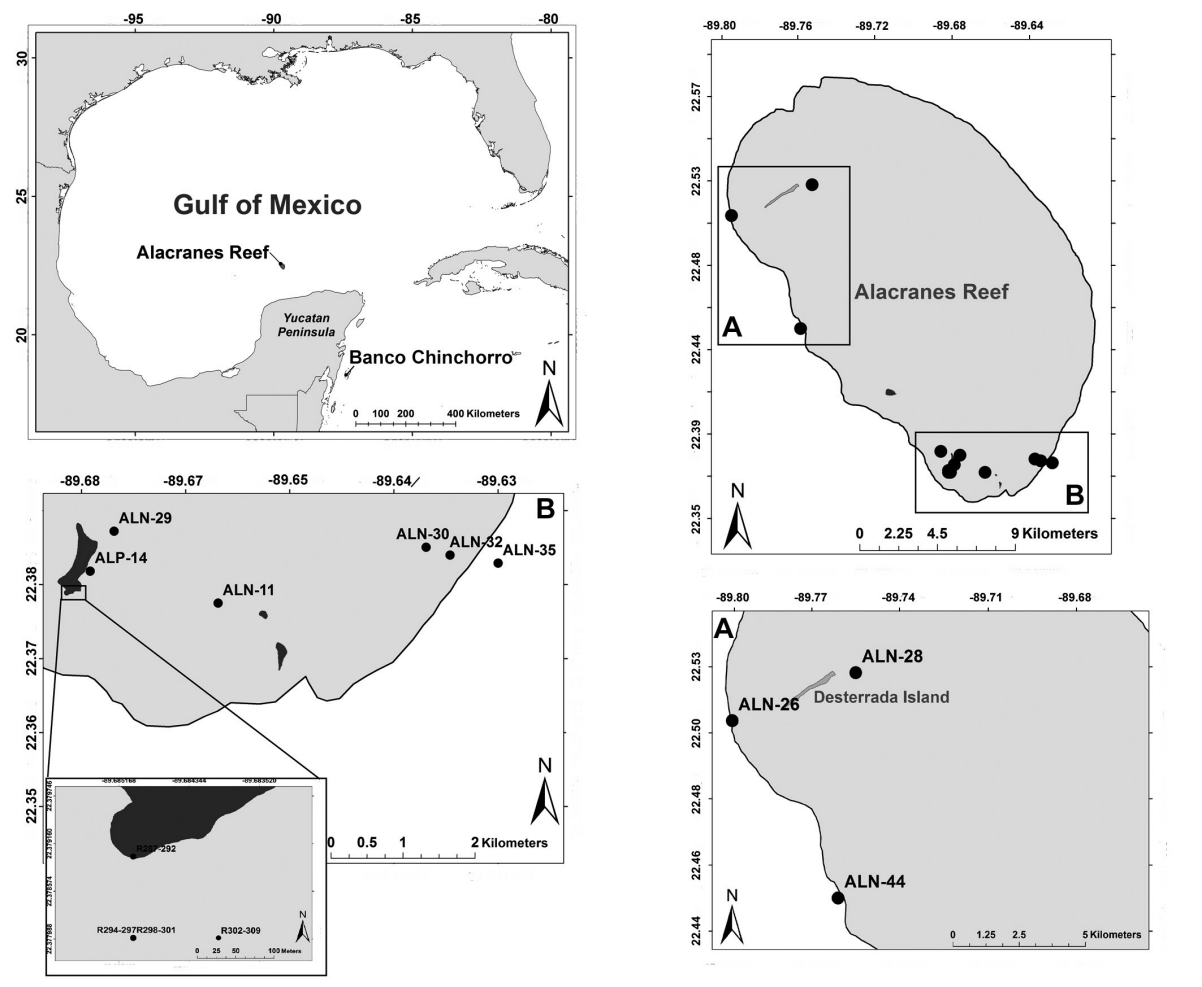
Location of the Arrecife Alacranes National Marine Park in the Gulf of Mexico. **A** General view of the sampling sites at the Alacranes reef **B** Southern area of the reef and Perez Island collecting sites.

Samples were obtained at PNAA reef (Figure 1) as part of a multidisciplinary biodiversity project during two surveys in May-June 2008 and July-August 2009 (Table 1). The chiton species were collected in the intertidal zone of Perez Island and in several different reef environments, by snorkeling and SCUBA, from the intertidal up to 12 m depth. Collecting protocol followed direct searching on reef structures and boundaries, as well as other hard substrates including wood, dead coral, and algae. All specimens collected were anesthetized in 8% clove oil or magnesium chloride, followed by preservation in 70–100% ethanol. The plates, girdle elements and radula were examined using a scanning electron microscope (SEM) following García-Ríos (2015) methodology for cleaning and coating the structures. The images were taken with a Hitachi SU1510.10.0kV SEM at the Instituto de Biología (IB), or with a JEOL JSM6360LV SEM at the Instituto de Ciencias del Mar (ICMyL), both part of the Universidad Nacional Autónoma de México (UNAM). The specimens were held and vouchered in the Colección Nacional de Moluscos (CNMO) at the Instituto de Biología, UNAM. Systematic arrangement follows Sirenko (2006), and Irisarri et al. (2014) for Acanthochitonina Bergenhayn, 1930.

**Table 1.** List of stations within the National Marine Park Arrecife Alacranes showing the georeferences and the depth in which the specimens were collected.

**Site**	**Latitude**	**Longitude**	**Date**	**Type of sampling**	**Depth**
R287-292	22.379	-89.685	02-Jun-2008	Intertidal (by hand)	0–1 m
R294-297	22.378	-89.685	03-Jun-2008	Intertidal (by hand)	0–1 m
R298-301	22.378	-89.685	04-Jun-2008	Intertidal (by hand)	0–1 m
R302-309	22.378	-89.684	05-Jun-2008	Intertidal (by hand)	0–1 m
ALN-11	22.378	-89.666	04-Aug-2009	Snorkel	1–2 m
ALN-14	22.389	-89.689	05-Aug-2009	Snorkel	1–2 m
ALN-26	22.512	-89.798	08-Aug-2009	Scuba	12 m
ALN-28	22.528	-89.756	08-Aug-2009	Scuba	12 m
ALN-29	22.387	-89.679	08-Aug-2009	Scuba	12 m
ALN-30	22.385	-89.640	09-Aug-2009	Scuba	12 m
ALN-31	22.384	-89.632	09-Aug-2009	Snorkel	1–2 m
ALN-32	22.384	-89.637	09-Aug-2009	Scuba	12 m
ALN-35	22.383	-89.631	10-Aug-2009	Scuba	12 m
ALN-44	22.453	-89.762	13-Aug-2009	Scuba	12 m
ALP-14	22.382	-89.682	08-Aug-2009	Intertidal (by hand)	0–1 m

## Results

A total of 58 specimens belonging to five families, seven genera, and nine species were found. The most diverse family was the Acanthochitonidae Pilsbry, 1893 with four species, followed by Ischnochitonidae Dall, 1889 with two, and Chitonidae Rafinesque, 1815, Callistoplacidae Pilsbry, 1893, and Lepidochitonidae Iredale, 1914 with one species each. *Tonicia schrammi* (Shuttleworth, 1856) and *Lepidochitona liozonis* (Dall & Simpson, 1901) are new records for the Gulf of Mexico or Caribbean coasts of Mexico (see Appendix).

*Ischnochiton erythronotus* (C. B. Adams, 1845), *Stenoplax bahamensis* Kaas & Van Belle, 1987, *Calloplax janeirensis* (Gray, 1828) and *Cryptoconchus floridanus* (Dall, 1889) were sampled in both surveys. In addition, the results of a literature review and present findings herein reported, increases to 21 species for the overall known diversity of chitons from the eastern coasts of Mexico (see Appendix).

## Systematics

### Class Polyplacophora Gray, 1821

#### Order Chitonida Thiele, 1910

##### Suborder Chitonina Thiele, 1910

###### Family Ischnochitonidae Dall, 1889

####### Genus *Ischnochiton* Gray, 1847

######## Ischnochiton erythronotus

Taxon classificationAnimaliaChitonidaIschnochitonidae

(C. B. Adams, 1845)

Figures 2A–H , 3A–G

######### Material examined.

20 specimens; 0.5–16 mm long, 0.3–8.5 mm wide. Laguna Arrecifal Desterrada (CNMO4980), Isla Perez (CNMO4981, CNMO4982, CNMO5002), Cabaña y Playa CONANP (CNMO4983, CNMO4984, CNMO4989, CNMO4998, CNMO5000, CNMO5003, CNMO5004), Cabezas entre Blanca y Pajaros (CNMO4985), Playa Norte (CNMO4986, CNMO4988, CNMO5001), Playa Arrecifal (CNMO4987).

######### Description.

**Figure 2.**
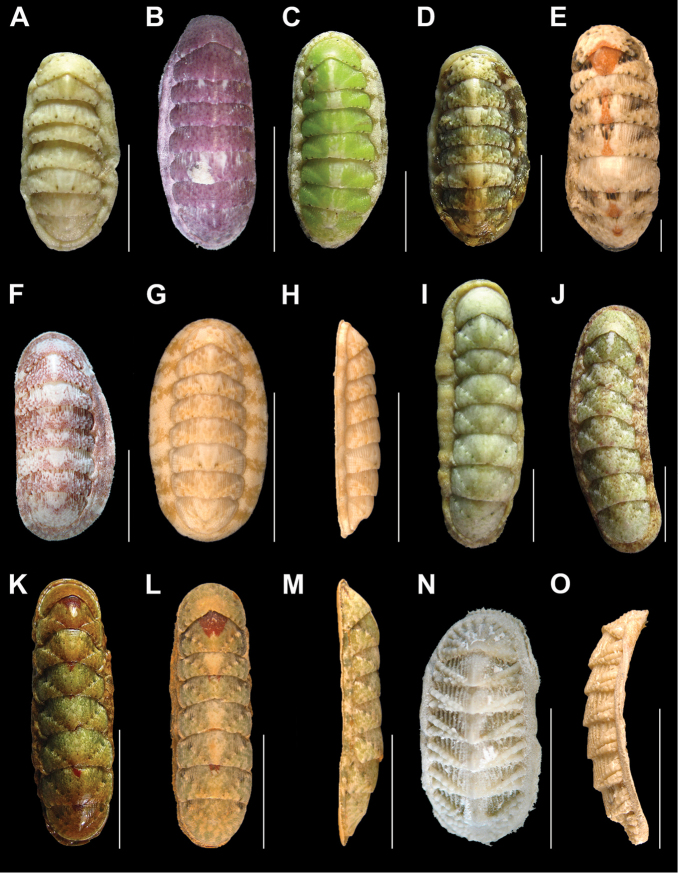
*Ischnochiton erythronotus* (C. B. Adams, 1845), **A–H**: in dorsal view, showing the tegmentum color variability **A** specimen from Laguna Arrecifal Desterrada (CNMO4980, 15.2 mm length) **B** specimen from Isla Perez (CNMO4981, 93.7 mm length) **C** specimen from Cabaña y Playa CONANP (CNMO4983, 11.7 mm length) and **D** (CNMO5003, 12.9 mm length) **E** specimen from Cabezas entre Blanca y Pajaros (CNMO4985, 13.6 mm length) **F** specimen from Playa Norte (CNMO4988, 14.2 mm length) **G** specimen from Playa Arrecifal (CNMO4987, 15.2 mm length length) and **H**, in lateral view. *Stenoplax bahamensis* Kaas & Van Belle, 1987 **I–M**: in dorsal view, showing the tegmentum color variability **I** specimen from Playa Norte (CNMO4942, CNMO4943, 22.1 mm length) **J** specimen from Pared Arrecifal (CNMO4957, 24.5 mm) **K** Laguna Desterrada (CNMO4974, 23.4 mm length), and **L** and **M**, in dorsal and lateral view (CNMO4974, 22.8 mm length). *Calloplax janeirensis* (Gray, 1828) **N** specimen from Isla Perez (CNMO4994, 12.5 mm length), in dorsal view and **O** (CNMO4990, 13.4 mm length), in lateral view, SB = 1 cm.

**Figure 3.**
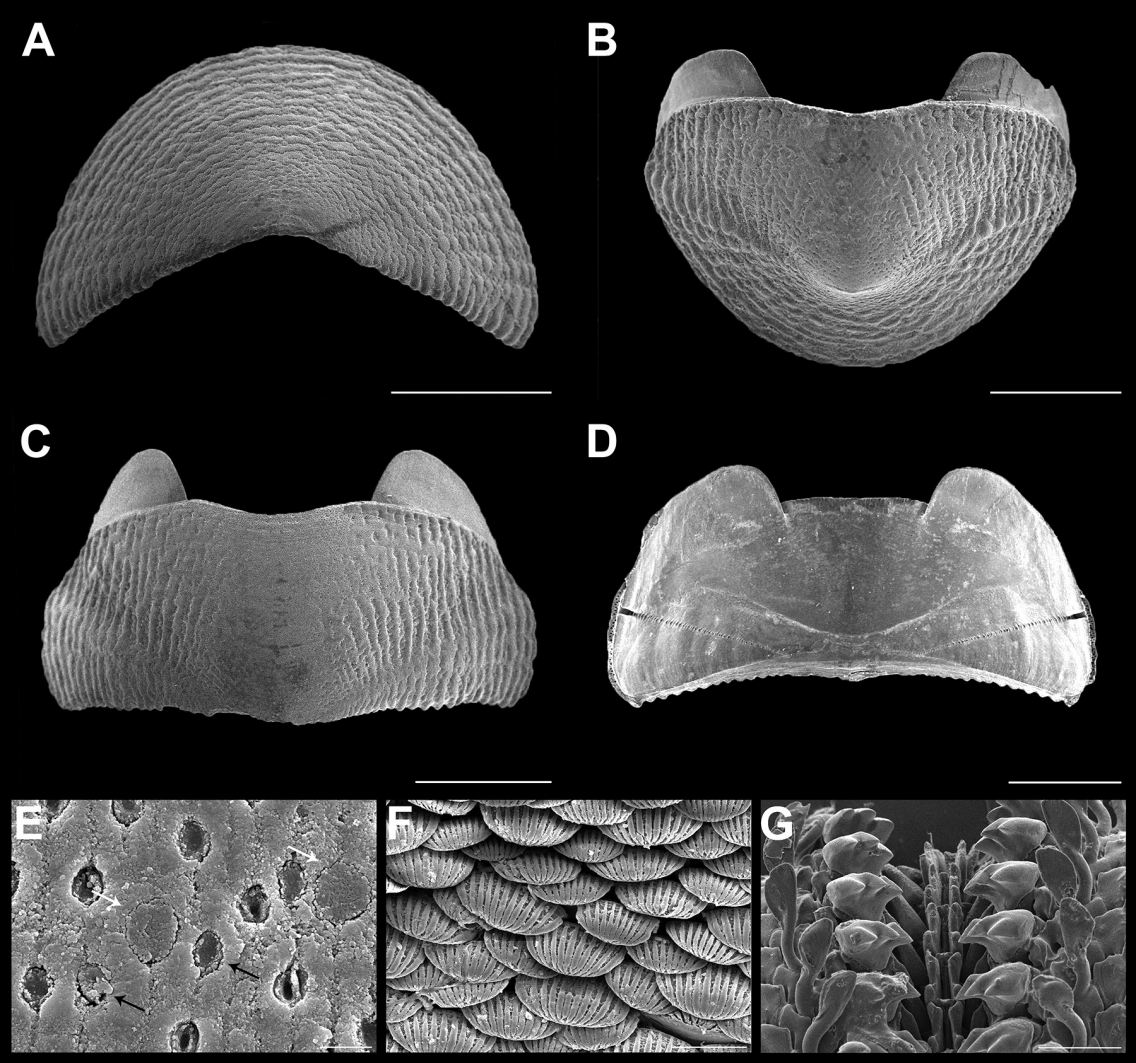
*Ischnochiton erythronotus* (C. B. Adams, 1845). Scanning electron images of a specimen from Laguna Arrecifal Desterrada (CNMO4980, 15.2 mm length); **A** head valve (I) in dorsal view, SB = 1 mm. **B** dorsal view of tail valve (VIII), SB = 1 mm **C** dorsal view of intermediate valve IV, SB = 1 mm **D** ventral view of intermediate valve IV, SB = 1 mm **E** detail of aesthete system, white arrows indicating the megalaesthetes, black arrows indicating the micraesthetes of intermediate valve IV, SB = 10 μm **F** girdle scales detail, SB = 100 μm **G** detail of radular teeth, SB = 100 μm.

Small-sized chitons, broad oval shape. Color of tegmentum and girdle very variable, mostly creamy, red, purple or light green and mottled with dark brown dots or patches (Figure 2A–H). Tegmentum sculptured with irregular concentric riblets and longitudinal narrow ribs. Head valve (Figure 3A), semicircular, not notched, sculpturing of irregular concentric ribs, broken into numerous riblets, forming fine radial indicated grooves. Tail valve (Figure 3B), wider than long, mucro postmedian, somewhat elevated; antemucronal area with 9–11 narrow, sometimes branched longitudinal narrow ribs; postmucronal area with concentric riblets and nodules, forming 19–23 radial grooves. Intermediate valves (Figure 3C), semi-rectangular outlined, side margins rounded and posterior margin from slightly concave to straight; lateral areas somewhat elevated, sculptured as the head valve, with two to three radial grooves; pleural areas with 12–14 longitudinal ribs, which fade towards the jugal area. Articulamentum laterally short; apophyses narrow, subtriangular shaped, jugal sinus wide and smooth (Figure 3D), slit formula 8–10/1/7–10. Megalaesthetes surrounded by 7–8 large micraesthetes (Figure 3E). Girdle variable in color as tegmentum, with alternating irregular bands of dark and lighter color, dorsally with wide, short scales (Figure 3F), with 10–12 wide and flat ribs. Radula (Figure 3G), with major lateral teeth tricuspid, the outer cusp is shorter than the others, central tooth very narrow anteriorly wider and spatulated.

######### Habitat.

Found in intertidal to shallow subtidal to 12 m depth. Found on dead coral, wood, rock, and sunken turtlegrass, *Thalassia testudinum* Banks ex König.

######### Remarks.

This species displays a considerable intraspecific variation in color and valve sculpturing; also observed in chitons from Cozumel Island and in Banco Chinchorro reef (in the most southern region of Quintana Roo) (CNMO5558), which was also noted by García-Ríos (2003) for specimens from Puerto Rico and Ferreira (1978a) for Jamaica specimens. This variability was found in both, the juvenile and adult chitons morphology. In general, specimens reaching a length from 8–12.1 mm showed more regular and less broken ribs, whereas animals between 13.5–16 mm length developed branched ribs, particularly in pleural areas, and occasionally showed nodule formations on the head and the postmucronal area of the tail valve. This is the most common and abundant chiton species in the PNAA.

####### Genus *Stenoplax* Carpenter MS, Dall, 1879

######## Stenoplax bahamensis

Taxon classificationAnimaliaChitonidaIschnochitonidae

Kaas & Van Belle, 1987

Figures 2I–M , 4A–H

######### Material examined.

15 specimens; 11–25.4 mm long, 3–8.1 mm wide. Playa Norte (CNMO4942, CNMO4956, CNMO4973, CNMO4976), Isla Perez (CNMO4943, CNMO4944, CNMO4977), Pared Arrecifal (CNMO4957), Blanca y Pajaros (CNMO4961), Laguna Desterrada (CNMO4974, CNMO4975), Laguna Arrecifal (CNMO4978).

######### Description.

**Figure 4.**
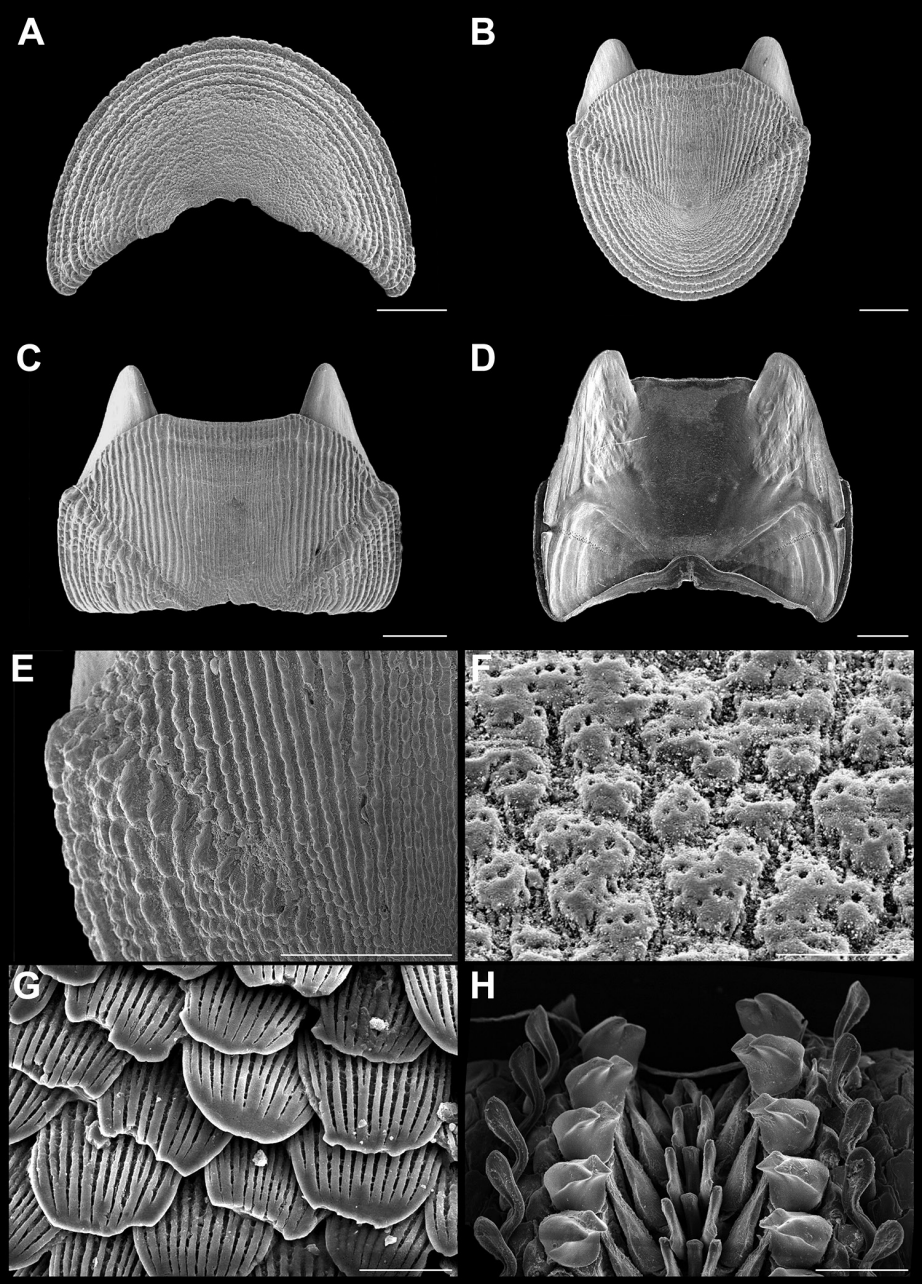
*Stenoplax bahamensis* Kaas & Van Belle, 1987. Scanning electron images of specimen from Playa Norte (CNMO4973, 23.6 mm length). **A** head valve (I) in dorsal view, SB = 1 mm **B** dorsal view of the tail valve (VIII), SB = 1 mm **C** dorsal view of intermediate valve IV, SB = 1 mm **D** ventral view of intermediate valve IV, SB = 1 mm **E** detail of intermediate valve IV, showing the adjacent sculpturing of the lateral and central areas, SB = 1 mm. **F** aesthetes detail of valve IV, SB = 100 μm **G** girdle scales detail, SB = 50 μm **H** detail of radular teeth, SB = 150 μm.

Medium-sized, elongate-oval chitons, around three times longer than wide. Color of tegmentum variable, mostly cream, dark and lighter green, light brown, with scattered dark brown spots (Figure 2I–M). Tegmentum with nodulose ribs, arranged concentric to the apex. Head valve (Figure 4A), semi-circular, slightly notched; sculpturing with nodulose ribs, break into numerous and regular nodules, particularly to the periphery. Tail valve (Figure 4B), with elevated postmedian mucro; postmucronal area sculptured as head valve, antemucronal area with 37–42 nodulose ribs. Intermediate valves (Figure 4C), with side margins somewhat rounded; lateral areas elevated, with 14–18 concentric nodulose ribs; pleural areas with 16–19 longitudinal ribs; the ribs next to lateral areas developed few small irregular to rounded lobules or pustules. Articulamentum slightly light blue; apophyses narrow and long twice as wide, wing shaped: jugal laminae smooth and wide (Figure 4D); slit formula 9–13/1/9–13. The ribs next to the postmucronal region tend to form more lobule or pustule like formations than intermediate valves (Figure 4E). Megalaesthetes small and surrounded by 4–6 smaller micraesthetes (Figure 4F). Girdle covered with tiny scales (Figure 4G), each with 11–13 wide, somewhat flat ribs on its surface. Radula (Figure 4H), with tricuspid major lateral tooth, cusps pointed and rather irregular in shape and length, the central and minor lateral teeth about the same length and as narrow and short spatulated shaped.

######### Habitat.

Found in intertidal to shallow subtidal to 12 m depth, on sunken wood, rock and turtlegrass, *T. testudinum*.

######### Remarks.

According to Arce and García-Ríos (2015), the sculpturing of small adults of *S. floridana* (18.6 mm) is very similar to *S. bahamensis* adults (31.8 mm), with somewhat continuous ribs in lateral areas. And the juvenile forms of both species (9.6 and 8.6 mm respectively) are almost identical, making their identification quite difficult.

PNAA *S. bahamensis* juveniles (ranging from 11–14.2 mm length), showed continuous ribs in lateral and central areas, with few granular formations next to the diagonal ridge; and our comparison with *S. floridana* was limited to one adult specimen.

Our observations of adult specimens agree with previous authors (Bullock 1985, Kaas and Van Belle 1987), that distinguish *S. bahamensis* from *S. floridana* by the absence of inconspicuous pustule formation next to the lateral area (Figure 4E). Examination of *S. floridana* (26.7 × 9.2mm) (CNMO5557) from Banco Chinchorro, Quintana Roo (Figure 14E–G), revealed central areas with granulate ribs, and lateral areas raised higher, with strong tuberculated discontinuous ribs, also present in head valve and postmucronal area of tail valve, whereas *S. bahamensis* develops lower lateral areas with more continuous ribs.

Bullock (1985) contributed on the knowledge of Caribbean *Stenoplax* s.s. species. He presented an exhaustive review of species aesthete density, major lateral tooth outline and sculpturing morphology. Based on his observations, he grouped *S. bahamensis* and *S. floridana* in a single lineage, defined on the reduced rib width, fewer aesthetes and elongate denticle cap. In addition to the examination, he also included the basal spot of the major lateral tooth, and an outline of the denticle cap, which according to him was distinctive, and useful as taxonomic character to distinguish among other chiton species. However, the differentiation of *Stenoplax* Caribbean species we have used here relies more on adult sculpturing differences (on how broken the ribs can appear, and if there is pustule development (Figure 14G) in the diagonal ridge of intermediate valves).

###### Family Callistoplacidae Pilsbry, 1893

####### Genus *Calloplax* Thiele, 1909

######## Calloplax janeirensis

Taxon classificationAnimaliaChitonidaCallistoplacidae

(Gray, 1828)

Figures 2N–O , 5A–H

######### Material examined.

Four specimens; 9–15.2 mm long, 4.9–7.1 mm wide. Isla Perez (CNMO4981, CNMO4990, CNMO4994).

######### Description.

**Figure 5.**
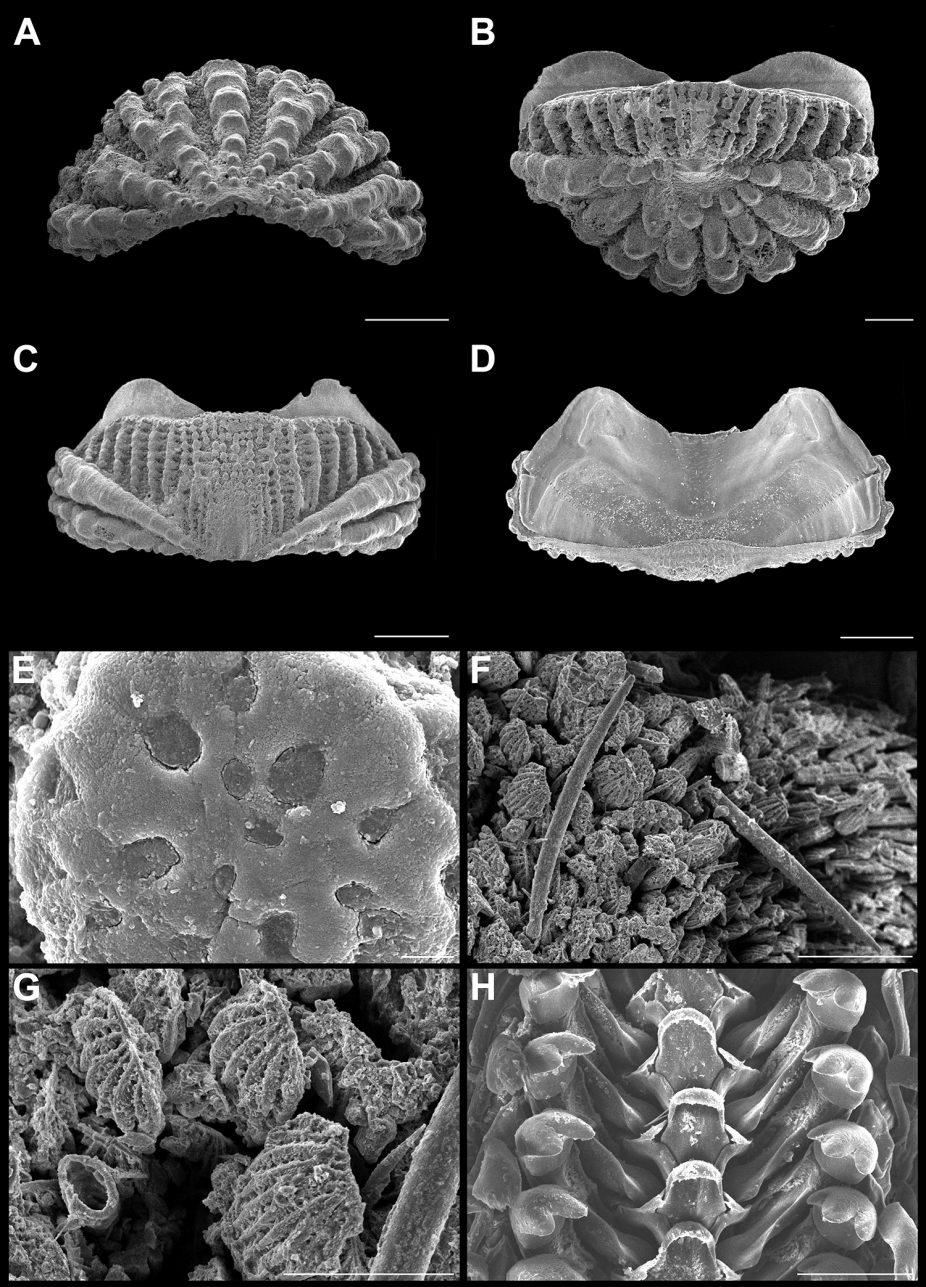
*Calloplax janeirensis* (Gray, 1828). Scanning electron images of a specimen from Isla Perez (CNMO4981, 13.9 mm length). **A** dorsal view of head valve (I), SB = 1 mm **B** dorsal view of tail valve (VIII), SB = 1 mm **C** dorsal view of intermediate valve IV, SB = 1 mm **D** ventral view of intermediate valve IV, SB = 1 mm **E** pustule detail with aesthetes, SB = 10 μm **F** girdle scales and hyaline needles, SB = 100 μm **G** detail of girdle spicules, SB = 50 μm **H** detail of radular teeth, SB = 100 μm.

Medium-sized chitons with elongate body shape. Color yellow, creamy, or light brown (Figure 2N–O). Tegmentum strongly sculptured with large, raised coalesced pustules. Head valve (Figure 5A), semi-circular, with large lobulose pustules, arranged in 8–10 radiating, bifurcated rows, uplifted notch. Tail valve (Figure 5B), wider than long; mucro postmedian, not elevated and pointed; antemucronal area with 17–19 longitudinal, pustulose ribs, the pustules on the jugal area are less coalesced and arranged longitudinally; postmucronal area with 8–9 coarsely pustulose radial ribs. Intermediate valves (Figure 5C), semi-rectangular shaped, posterior margin almost straight; lateral areas heavily elevated with 2–3 coarsely pustulose bifurcate ribs; the rib adjacent to central area usually raised more than those near the posterior margin; central areas narrower than combined lateral areas, wider near its center; pleural areas with 7–8 longitudinal ribs, which can develop sub-riblets; 5–7 pustulose ribs in jugal areas, which fade toward the apex. White to slightly blue articulamentum; apophyses and insertion plates short and wide (Figure 5D); slit formula 7–9/1/8–10. Pustules bear large megalaesthetes and between them, the micraesthetes appear somewhat scattered (Figure 5E). Girdle colored in alternating irregular bands of green, cream and yellow; covered with small and medium strong ribbed scales, and hyaline long needles (Figure 5F), the scales are somewhat wider than longer, its apical end develop a flattened pit (Figure 5G), and between the scales occur single curve spicules, scattered with no apparent order. Radula (Figure 5H) with major lateral tooth tridentate, with broad and wide cusps; central tooth spatulate in shape, its distal end bending outwards and longer than minor lateral teeth.

######### Habitat.

Found in intertidal, on rock buried in sand, associated with crustose coralline red algae.

######### Remarks.

When Thiele (1909) described the genus *Calloplax* based on only its type species, *Chiton janeirensis* Gray 1828, he emphasized differences of its tegmental nodulose ribs contrasted with those of the distinct pustules for selected members of *Chaetopleura* Shuttleworth, 1853, where Pilsbry (1892) had previously assigned this species. Smith and Ferreira (1977), Ferreira (1978b) and Ferreira (1982) expanded Thiele’s genus to include three other species, with Ferreira (1978b) transferring it from Chaetopleuridae to Callistochitonidae, and then later (1982) transferring it back. Ferreira (1978b) designated and figured a lectotype from among the syntypes of *Chiton janeirensis* from the type locality of Rio de Janeiro, Brazil. Lyons (1985) questioned the reassignment of family status for *Calloplax janeirensis*, emphasizing the morphological similarities between *Calloplax* and *Chaetopleura* genera, also noted by Ferreira (1978b; 1982). Lyons (1985) acknowledged the uncertainty regarding both genera, and based on similar girdle elements and the continuity in the range of tegmental sculpturing in species assigned to *Chaetopleura* or *Calloplax*, he suggested that *Calloplax* should be considered a synonym of *Chaetopleura* s.s. This was later followed by Bullock et al. (1994) and Lyons and Moretzsohn (2009), but Van Belle (1983), Kaas and Van Belle (1994; 1998) and Sirenko (2006) recognized it as a separate genus and have followed Ferreira (1978b) in placing it in a subfamily or family, respectively, associated with *Callistochiton*, not *Chaetopleura*, based on the presence of heavily sculptured ribs, and the insertion plate slits that generally correspond in number and position to the dorsal radial ribs, which, according to these authors is not a state of character of Chaetopleurinae. Recognizing that its status remains controversial, here we have preferred to follow Sirenko (2006) for the assignment of *Calloplax* as a distinct genus within Callistochitonidae.

PNAA specimens (up to 15.2 mm), bear resemblance on sculpturing and number of ribs (I: 8 ribs; IV 2–3 ribs; VIII 9 ribs) to a 10.5 mm specimen of *C. janeirensis* described by Lyons (1985: figs 22–24) from Dry Tortugas, Florida Keys, for which he figured valves I (11 ribs), IV (2 ribs) and VIII (8 ribs). PNAA specimens show ribs that tend to merge and bifurcate, and the lateral areas seem to develop three pustulose ribs, which became two, after both ribs next to diagonal ridge merged into one. On the contrary, Florida Keys specimens show more rounded pustules, the ribs remain separate or well-defined, and the lateral areas display two separated ribs. The number of ribs on the pleural areas (8 ribs), antemucronal area (18 ribs) and postmucronal areas of tail valve (8 ribs), are similar in both species. Such differences between Florida Keys and PNAA can be explained by the chiton’s size. We assume that Lyons (1985) described a juvenile specimen. Examination of the lectotype (NHMUK 1977041/2) from Rio de Janeiro (16.2 mm) (Figure 14C), showed rounded pustules aligned in ribs, 12 on head valve, 3–4 on lateral areas, and 15 ribs on postmucronal area of tail valve. When comparing with the PNAA specimen (15.2 mm), the lectotype has no fusion between pustules and it has a higher number of ribs on the head valve, lateral and postmucronal areas. The pustule morphology and number of ribs, of the lectotype is similar to specimens that García-Ríos (2003: fig. 77–81) figured from Puerto Rico. Overall, the PNAA specimens showed similarities with previously figured specimens from the Bahamas, Florida and Puerto Rico in the girdle scales and spicules, and in the morphology of the major lateral radula teeth (Ferreira 1978b, Lyons 1985, Kaas and Van Belle 1994, García-Ríos 2003).

###### Family Chitonidae Rafinesque, 1815

####### Subfamily Toniciinae Pilsbry, 1893

######## Genus *Tonicia* Gray, 1847

######### Tonicia schrammi

Taxon classificationAnimaliaChitonidaChitonidae

(Shuttleworth, 1856)

Figures 6A–C , 7A–K

########## Material examined.

Two specimens; 27.5 and 28.5 mm long, 14 and 14.2 mm wide. Cabaña CONANP (CNMO4992).

########## Description.

**Figure 6.**
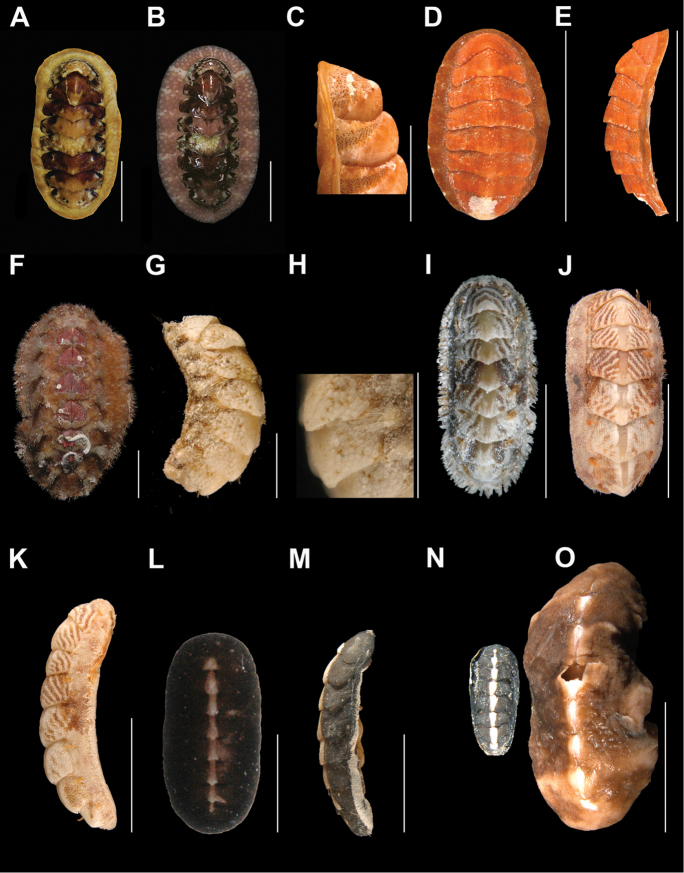
*Tonicia schrammi* (Shuttleworth, 1856), **A** preserved specimen, showing the color change **B** life specimen coloration from Cabaña CONANP (CNMO4992, 28.5 mm length) **C** same specimen in lateral view, SB = 1 cm. *Lepidochitona liozonis* (Dall & Simpson, 1901) **D** dorsal view and **E** lateral view of a specimen from Isla Perez (CNMO5002, 10 mm), SB = 1 cm. *Acanthochitona hemphilli* (Pilsbry, 1893) **F** dorsal view of a specimen from Laguna Arrecifal (CNMO4997, 34.8 mm length), SB = 1 cm. *Acanthochitona roseojugum* Lyons, 1988 **G** in dorsal view and **H** detail of valve IV and V in lateral view, of specimen from Isla Perez (CNMO4995, 3 mm length), SB = 1 mm. *Acanthochitona zebra* Lyons, 1988 **I** dorsal view of a life specimen **J** same specimen in preserved conditions **K** in lateral view of same specimen from Cabaña CONANP (CNMO4979, 9.2 mm length), SB = 5 mm. *Cryptoconchus floridanus* (Dall, 1889) **L** dorsal view of life specimen **M** lateral view of same specimen under preserved conditions from Isla Perez (CNMO4996, 9.3 mm length), SB = 5 mm **N** (CNMO4996) and **O** (CNMO5560, 20.3 × 8.3 mm length, Banco Chinchorro, Quintana Roo), shows the length comparison, SB = 1 cm.

**Figure 7.**
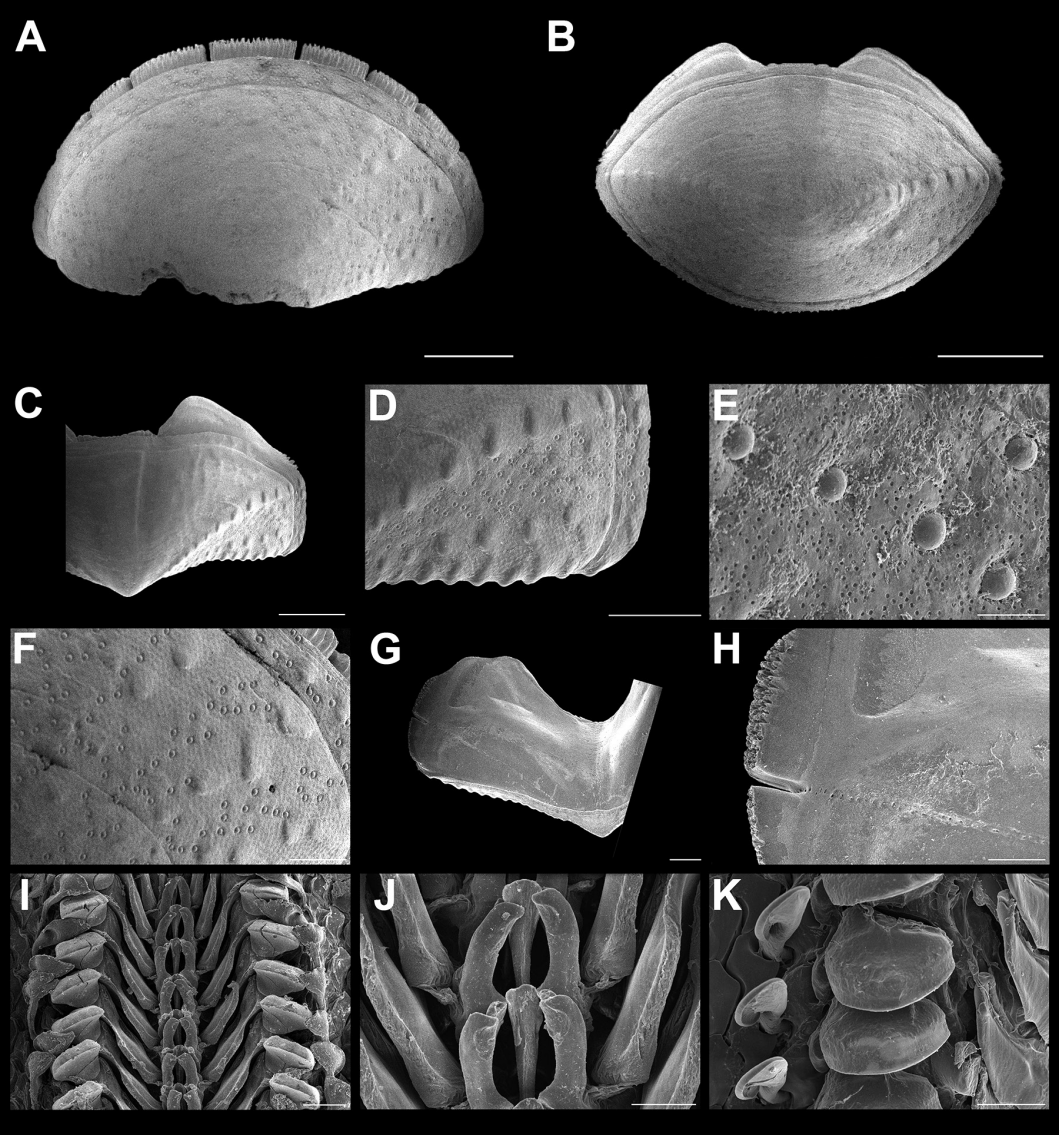
*Tonicia schrammi* (Shuttleworth, 1856). Scanning electron images of a specimen from Cabaña CONANP (CNMO4992, 27.5 mm length). **A** dorsal view of head valve (I), SB = 1 mm **B** dorsal view of tail valve (VIII), SB = 1 mm **C** dorsal view of intermediate valve IV, SB = 2 mm **D** detail of lateral area in dorsal view of the intermediate valve IV, SB = 1 mm **E** lateral area ocelli detail of intermediate valve IV, SB = 100 μm **F** head valve (I) detail, showing the ocelli alignment, SB = 50 μm. G, ventral view of intermediate valve IV, SB = 1 mm **H** insertion teeth and slit detail of intermediate valve IV in ventral view, SB = 50 μm **I** radular teeth, SB = 100 μm **J** central and minor lateral teeth detail, SB = 10 μm **K** major lateral teeth detail, SB = 50 μm.

Medium-sized chitons with an oval outline. Live specimens mostly pink, dark purple and white (Figure 6B), turning to orange when preserved; creamy with large dark brown spots (Figure 6A, C); girdle with narrow white bands, and rounded small spots scattered without any clear pattern. Head valve (Figure 7A) semicircular and slightly notched; sculpture pattern smooth, with fainted radial irregular knobs. Tail valve (Figure 7B) oval; mucro elevated and slightly postmedian; postmucronal area bear few knobs radially oriented to the mucro; antemucronal area smooth. Intermediate valves (Figure 7C) with strongly bluntly pointed apex. Lateral areas (Figure 7D) strongly indicated by a diagonal rib of semi rectangular knobs, also present on the posterior margin, which gives the appearance of dentations, and in between them a third rib with fewer knobs that are closer to the posterior margin . Ocelli arranged in irregular radial bands. Micraesthetes small, grouped with no apparent number and arrangement, others seem to be scattered (Figure 7E). Tegmentum with scattered small black rounded ocelli, forming radial irregular bands (Figure 7F). Girdle covered with tiny spicules. Apophyses short and wide, semi triangular shaped (Figure 7G). Articulamentum white and thick, insertion teeth hardly dentate, slit deep (Figure 7H); slit formula 9–10/1/10–13. Radula (Figure 7I) with central teeth long and distally narrow, minor lateral tooth somewhat arched, long, smaller than central tooth, distally somewhat pointed (Figure 7J); major lateral tooth as one single, rounded, wide plate, not dentate (Figure 7K).

########## Habitat.

Found in the intertidal on rocks and turtlegrass, *T. testudinum*.

########## Remarks.

García-Ríos (2003: fig. 109) described Puerto Rico specimens, and figured the radula microstructure of a 21 mm specimen, which showed the medium tooth narrow and bent outward on its posterior end, and the minor lateral teeth somewhat straight. Our examination of a PNAA specimen of 28.5 mm length revealed the minor lateral tooth arched inwards, and the central tooth is rounded on its distal end (Figure 7J). Microstructure examination of PNAA specimen (28.5 mm) showed the ocelli aligned in irregular bans, somewhat in quincunx towards to the apex, and less abundant than the previous descriptions (Figure 7D, F).

The examination of one paralectotype (NMBE19115/5a) (Figure 14D), revealed a higher density of ocelli somewhat aligned in groups on the lateral areas, head valve and postmucronal area of tail valve. Kaas et al. (2006) described a specimen from Puerto Rico (La Paguera, Media Luna) with similar ocelli arrangement pattern and density. Our specimens showed less density of ocelli and although they appear to be aligned, their arrangement is more irregular (Figure 7D, F). The knowledge of this species in the Mexican Caribbean is limited, and only further studies will corroborate the morphologic distinctions of specimens from this area. In this study, we extend the distribution range of *T. schrammi* to the PNAA.

##### Suborder Acantochitonina Bergenhayn, 1930

###### Family Lepidochitonidae Iredale, 1914

####### Genus *Lepidochitona* Gray, 1821

######## Lepidochitona liozonis

Taxon classificationAnimaliaChitonidaLepidochitonidae

(Dall & Simpson, 1901)

Figures 6D–E , 8A–I

######### Material examined.

One specimen of 10 mm long, 6 mm wide. Isla Perez (CNMO5002).

######### Description.

**Figure 8.**
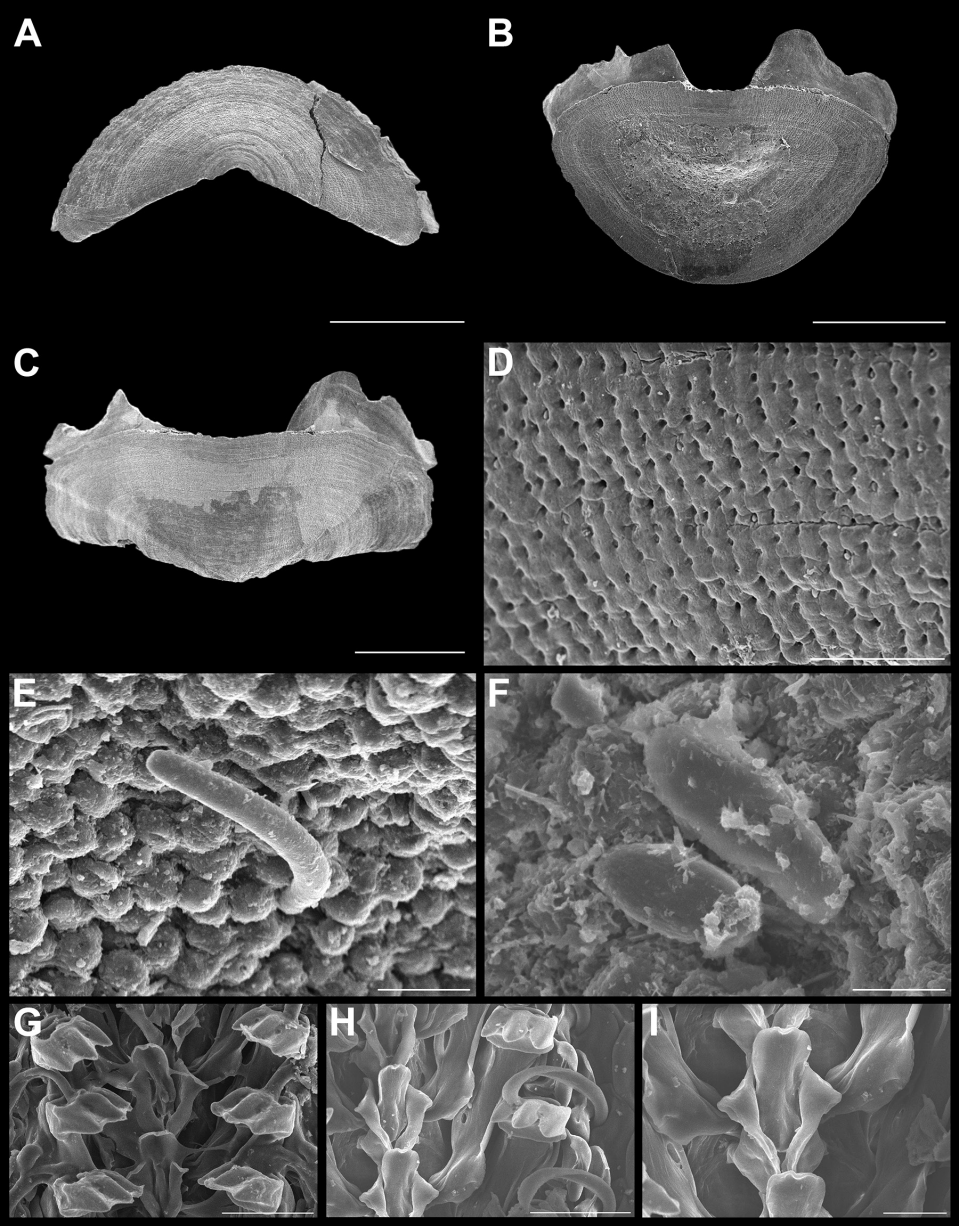
*Lepidochitona liozonis* (Dall & Simpson, 1901). Scanning electron images of a specimen from Isla Perez (CNMO5002, 10 mm length). **A** dorsal view of head valve (I), SB = 1 mm **B** dorsal view of tail valve (VIII), SB = 1 mm **C** dorsal view of intermediate valve IV, SB = 1 mm **D** detail of aesthetes of valve IV, SB = 100 μm **E** girdle hyaline spicules, SB = 20 μm **F** detail of girdle spicules, SB = 10 μm **G** radular teeth, SB = 50 μm **H** major lateral teeth detail, SB = 50 μm **I** detail of central and minor lateral teeth, SB = 20 μm.

Small-sized chiton, of elongate-oval shape. Tegmentum dark orange, mottled with small white spots, tail valve showing a large irregularly shaped white spot on the postmucronal area; girdle with irregular lighter and darker longitudinal bands (Figure 6D). Tegmentum micro-granular, smooth, usually with growth lines. Head valve (Figure 8A) semicircular; posterior margin “V” shaped and notched. Tail valve (Figure 8B), wider than long, semicircular, the antemucronal and postmucronal area are indicated by a weak diagonal ridge; mucro somewhat elevated, antemedian; postmucronal slope slightly concave. Intermediate valves (Figure 8C) broadly rectangular, side margins somewhat rounded, posterior margin convex, with a prominent beak; lateral areas not rosy, with a faint diagonal ridge line. Articulamentum thin and translucent; apophyses well separated, long and narrow; slit formula 10/1/12. Megalaesthetes large (Figure 8D), arranged longitudinally, forming a depression on the tegmentum. Girdle covered with small, short, distally rounded spicules (Figure 8E, F), scattered with hyaline curved spicules (Figure 8E), arranged in groups of 2–3. Radula (Figure 8G) with a long, spatulate central tooth, distally wider, with its anterior end curved outwards; major lateral tooth tricuspid, the cusps around the same size (Figure 8H), the minor lateral tooth sub triangularly shaped, almost half the size of the central tooth (Figure 8I).

######### Habitat.

Found in the intertidal on rocks, associated with crustose coralline red and brown algae.

######### Remarks.

This species was considered by Ferreira (1985) as a synonym of *Lepidochitona beanii* (Carpenter, 1857). According to him, there were no particular differences between similar appearing specimens from the Caribbean or Eastern Pacific. However, in our opinion, there are major differences between them. As earlier noted by Kaas and Van Belle (1985), *L. liozonis* has an antemedian mucro, the apophyses are sinuated and somewhat elongated, and the girdle shows irregular slender spicules. In contrast, *L. beanii* bears a postmedian mucro, has shorter and semi-rectangular shaped apophyses, and hyaline long spicules interspersed and bunched at the sutures in groups of 3–4.

Recently, García-Ríos (2015) compared the morphologic features and DNA sequences (mitochondrial cytochrome oxidase subunit I or COI) of *L. liozonis* from its type locality (Puerto Rico, Culebra Island, Ensenada Honda) to specimens from Florida Keys, which were considered as variety “*tropica*” (Pilsbry 1940). His results showed that these have to be considered as two different species. *Lepidochitona pseudoliozonis* García-Ríos, 2015 from Florida Keys is characterized by its larger body size (average of 9.7 mm), the deep concave postmucronal slope, and the longer marginal spicules. The Puerto Rico specimens (representing *L. liozonis*) can be distinguished by their smaller body length (average of 7 mm), the postmucronal slope being almost straight to somewhat concave, and with shorter marginal spicules. The examination of the *L. liozonis* type specimen (USNM161920) (Figure 14B) revealed that the postmucronal slope of the tail valve is somewhat concave (Figure 6E), and the intermediate valves are carinated with a pointed apex. The PNAA specimens resemble the holotype and the specimens from Puerto Rico, both with a slightly concave postmucronal slope, which seems to be a morphologic feature that discriminates both species.

###### Family Acanthochitonidae Pilsbry, 1893

####### Genus *Acanthochitona* Gray, 1821

######## Acanthochitona hemphilli

Taxon classificationAnimaliaChitonidaAcanthochitonidae

(Pilsbry, 1893)

Figures 6F , 9A–K

######### Material examined.

Seven specimens; 7–38 mm long, 3.2–21 mm wide. Agregacion Meros (CNMO4939, CNMO4999), Cabezas entre Blanca y Pajaros (CNMO4940), Yate Acatl (CNMO4946), Precio Caribbean (CNMO4941), Laguna Arrecifal (CNMO4997).

######### Description.

**Figure 9.**
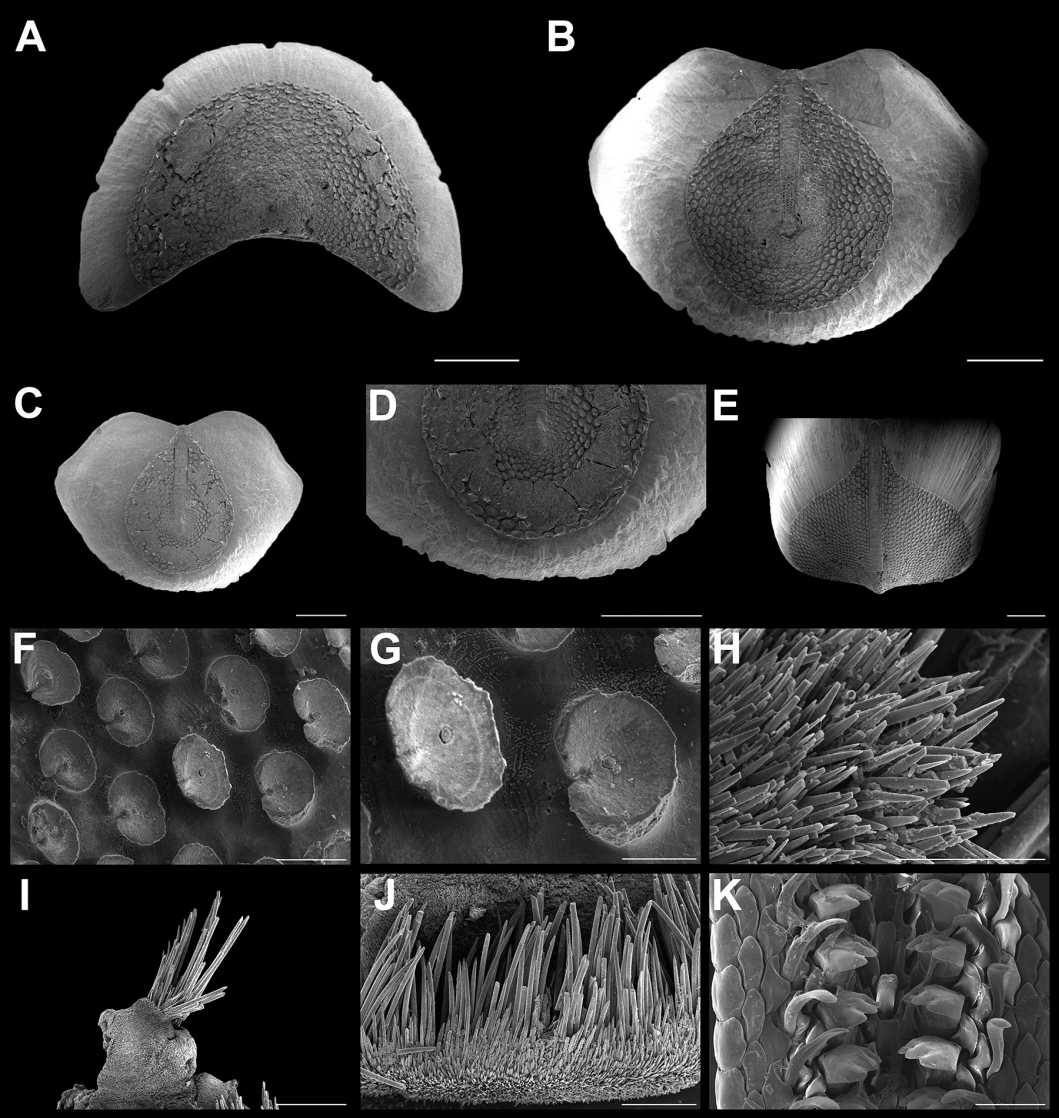
*Acanthochitona hemphilli* (Pilsbry, 1893). Scanning electron images of a specimen from Agregacion Meros (CNMO4939, 36.5 mm length). **A** dorsal view of head valve (I), SB = 1 mm **B** dorsal view of tail valve (VIII), SB = 1 mm **C** dorsal view of tail valve (VIII), of specimen from Laguna Arrecifal (CNMO4997, 38 mm length), SB = 1 mm **D** detail of articulamentum of tail valve (CNMO4997), SB = 1 mm **E** dorsal view of intermediate valve IV (CNMO4939), SB = 1 mm **F** pustules detail of tail valve (VIII), SB = 100 μm **G** detail of pustules of intermediate valve IV, SB = 50 μm **H** detail of girdle spicules, SB = 100 μm **I** detail of girdle tuft, SB = 1 mm **J** detail of ventral spicules of girdle, SB = 0.50 mm **K** radular teeth, showing central and major lateral teeth, SB = 200 μm.

Large chitons, body of oval shape. Tegmentum mostly red and orange (Figure 6F). Head valve (Figure 9A) semi-rounded, posterior margin “V” shaped, with concave apex, not notched; the pustule formations fade towards apex. Tail valve tegmentum drop shaped (Figure 9B); the pustules arranged concentrically; the postmedian somewhat elevated mucro not pointed (Figure 9B, C). Articulamentum solid, wide, well extending in all valves; wing-like shaped in the intermediate valves and the tail valve, with the slits short like a little opening and located posterior in the tail valve (Figure 9D); slit formula 5/1/2–1. Intermediate valves (Figure 9E) with rounded sides; jugal area narrow, with a pointed apex. Sculpture of semi-reniform pustules, more oval than elongated, with one single aesthete located in the center of the pustule (Figure 9F, G). Girdle wide and covered with small, hyaline smooth spicules (Figure 9H). Ventral spicules long and somewhat curved (Figure 9J). Dorsal tufts with long hyaline spicules (Figure 9I). Radula (Figure 9K) with a tridentate mayor lateral tooth, cusps rounded, the central cusp a little longer than the outer ones; the central tooth of rectangular shape, spatulate, longer than the minor lateral tooth.

######### Habitat.

Found from the intertidal to the shallow subtidal down to 12 m on rocks associated with crustose coralline red algae.

######### Remarks.

The PNAA specimens have more rounded or kidney-shaped pustules that are distributed across all valves (Figure 9F). The slits are shallow or not deep in the intermediate valves. In the tail valve, one of them is regularly absent (Figure 9D). The lack of the second slit on the tail valve was observed in three specimens, although there was no indication of any damage to the girdle or the valves, which sometimes may cause deformities in the articulamentum development. The central tooth of the PNAA species is a long sub rectangular plate, which in contrast to specimens from Puerto Rico (García-Ríos 2003: figure 133), show a conspicuously elongate-rounded tooth.

######## Acanthochitona roseojugum

Taxon classificationAnimaliaChitonidaAcanthochitonidae

Lyons, 1988

**Figure 10.**
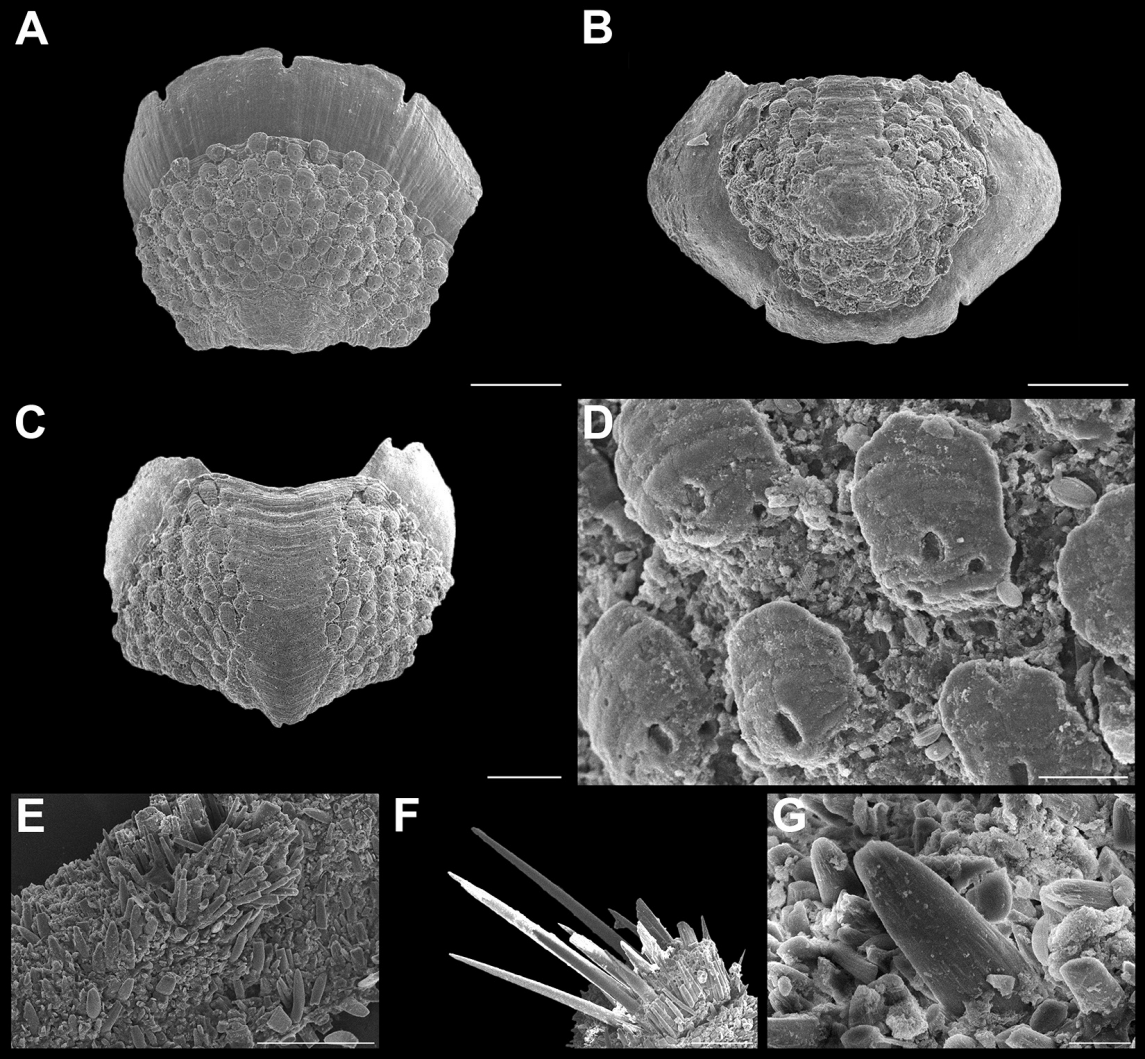
*Acanthochitona roseojugum* Lyons, 1988. Scanning electron images of a specimen from Isla Perez (CNMO4995, 3 mm length). **A** Dorsal view of head valve (I), SB = 200 μm **B** dorsal view of tail valve (VIII), SB = 200 μm **C** dorsal view of intermediate valve IV, SB = 200 μm **D** detail of pustules of intermediate valve IV, SB = 20 μm **E** girdle spicules, SB = 100 μm **F** girdle tuft detail, SB = 100 μm **G** detail of girdle spicules, SB = 10 μm.

Figures 6G–H , 10A–G

######### Material examined.

One juvenile specimen; 3 mm long, 1 mm wide. Isla Perez (CNMO4995).

######### Description.

Small-sized chiton, of a broad oval shape; tegmentum creamy color, with dark and lighter brown small spots; girdle irregularly banded in olive green and white (Figure 6G, H). Valves arched, somewhat elevated, especially the tail valve. Head valve (Figure 10A) wider than long, posterior margin almost straight; pustules directed radially towards the apex, apex smooth. Tail valve wider than long; mucro elevated and shifted somewhat postmedian (Figure 10B). Intermediate valves (Figure 10C) with a wide, smooth jugum, anteriorly somewhat straight; apex strongly pointed; pustules directed radially towards the apex. Articulamentum wide, especially on the tail valve, apophyses wing-shaped; slit formula 5/1/2. Tegmentum with sub-spatulate elongate pustules, with rounded edges, one single megalaesthete and two micraesthetes located at the pustule base (Figure 10D). Girdle covered with short and longer spicules (Figure 10E) and tufts with hyaline long needles (Figure 10F). The spicules are wider on the base and narrowing anteriorly; its apical area with a fine thin longitudinal striate (Figure 10G).

######### Habitat.

Found in the shallow subtidal at 12 m on rocks, associated with crustose algae.

######### Remarks.

The PNAA specimen of *A. roseojugum* display a single sub central megaesthete and two micraesthetes near the pustule base, which coincide with the description of Lyons (1988). Lyons also remarked this species high similarity in the sculpture pattern with *A. andersoni* Watters, 1981, and he found it difficult to separate these species. He called the few characters he established to distinguish the species from each other “subjective”. We found Lyons’ separation of these species to rely on only slight differences of pustule and valve morphology and body length. A more focused study is needed to clarify whether these nominal species are actually distinct species.

######## Acanthochitona zebra

Taxon classificationAnimaliaChitonidaAcanthochitonidae

Lyons, 1988

**Figure 11.**
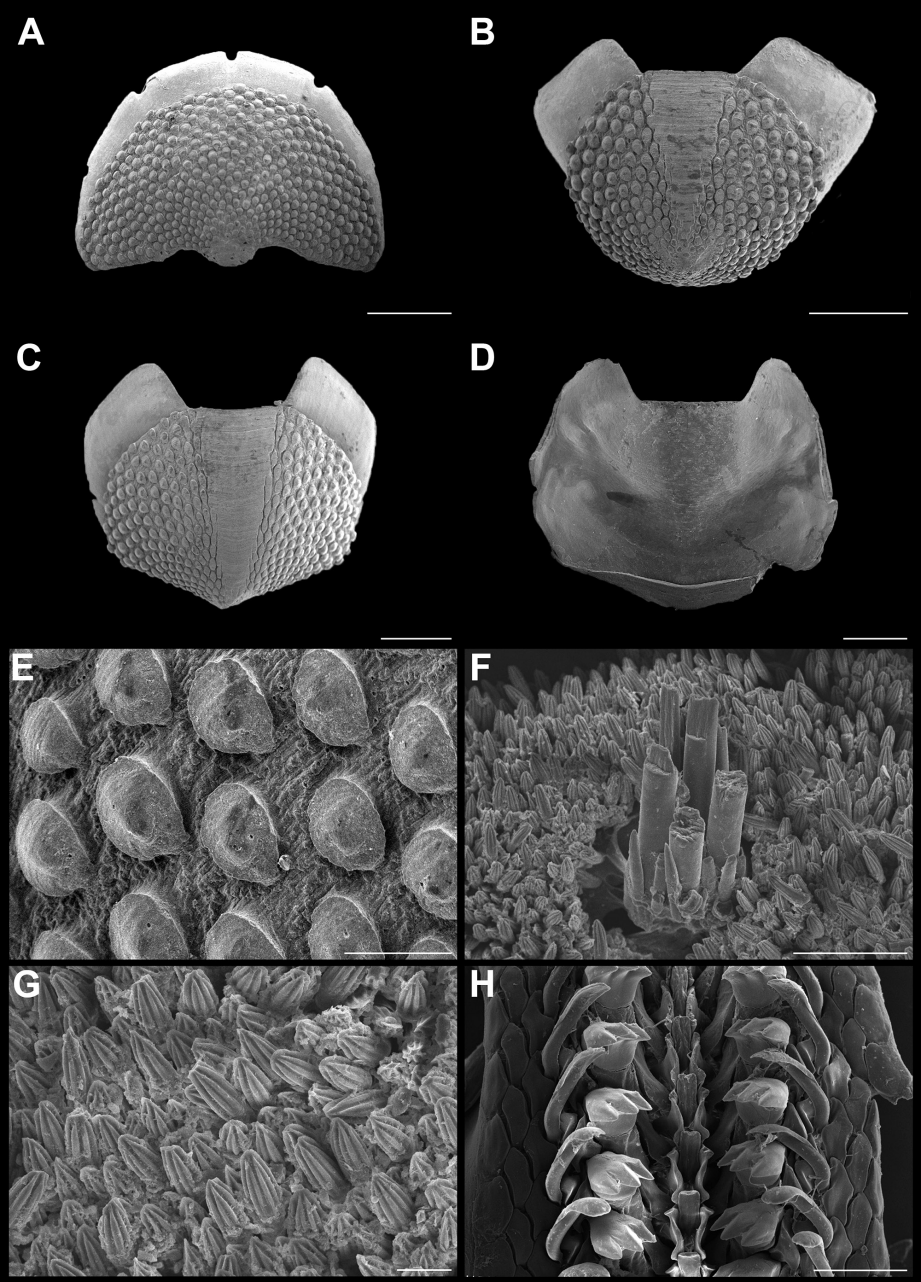
*Acanthochitona zebra* Lyons, 1988. Scanning electron images of a specimen from Laguna Desterrada (CNMO4993, 9.7 mm length). **A** dorsal view of head valve (I), SB = 500 μm **B** dorsal view of tail valve (VIII), SB = 500 μm **C** dorsal view of intermediate valve IV, SB = 500 μm **D** ventral view of intermediate IV, SB = 500 μm **E** intermediate valve IV pustules detail, SB = 100 μm **F** girdle spicules and tuft, SB = 100 μm **G** girdle spicules detail, SB = 20 μm **H** radular teeth, SB = 100 μm.

Figures 6I–K , 11A–H

######### Material examined.

Five specimens; 7.5–11.5 mm long, 3.2–7 mm wide. Laguna Arrecifal Desterrada (CNMO4945), Cabaña CONANP (CNMO4979), Laguna Desterrada (CNMO4993).

######### Description.

Small-sized chitons, color mainly beige to creamy, with concentric bands in dark brown or green on head, tail valve and the lateropleural areas of intermediate valves (Figure 6I–K). Head valve (Figure 11A) semicircular, wider than long, posterior margin straight, apex slightly present. Tail valve (Figure 11B) smaller than head valve; mucro postmedian. Intermediate valves (Figure 11C) with tegmentum wider than long; apex pointy; jugum smooth and wider anteriorly. Insertion plate curved, short on its sides; apophyses long and anteriorly elevated wing-shaped in intermediate valves (Figure 11D); sub-rectangular in tail valve; slits in intermediate valves and in the tail valve are hardly present; slit formula 5/1/2. Tegmentum covered with sub-spatulate pustules, wider on their posterior end and somewhat inflated on their central area (Figure 11E). Girdle in living specimens wide, mostly cream-white colored with dark brown or olive-green, irregular longitudinal bands; covered with small spicules (Figure 11F, G), flattened, elongated and strongly ribbed (7–8 ribs) spicules, the ribs reach and join the spicule apex (Figure 11G); tufts reduced or less dense, with long hyaline spicules (Figure 11F). Radula (Figure 11H) with a pointed tricuspid major lateral tooth, the cusps are of almost the same size; central teeth sub-rectangular or spatulate anteriorly curved outwards.

######### Habitat.

Found from the intertidal to the shallow subtidal around 12 m, on living corals, rocks and turtlegrass, *T. testudinum*.

######### Remarks.

Lyons (1988) described morphological variation in this species (figure 118–120, and 121–127 respectively) from Tamarind Beach reef in Grand Bahamas, Dry Tortugas Florida and Puerto Rico. According to those figures, it seems that the Bahamas specimen (figure 118–120, of 10 mm length) showed a wider tegmentum in IV, and a remarkably elongated head valve. A PNAA specimen (11.2 mm) showed similarities with those specimens from Florida (Lyons 1988: figure 125, 11 mm), particularly in its rounded tegmentum, which seems to be typical in specimens of nearly the same length, and which is also similar to the type specimen (USNM859319) (15 mm). This specimen is also characterized by a somewhat rounded outline of the tegmentum (Figure 14A). Closer examination of the pustules of the PNAA specimens revealed them to have a somewhat inflated surface on their center (Figure 11E). Lyons (1988) did not call attention to this character state but the pustules are similar in his figures.

####### Genus *Cryptoconchus* Blainville MS, Burrow, 1815

######## Cryptoconchus floridanus

Taxon classificationAnimaliaChitonidaAcanthochitonidae

(Dall, 1889)

**Figure 12.**
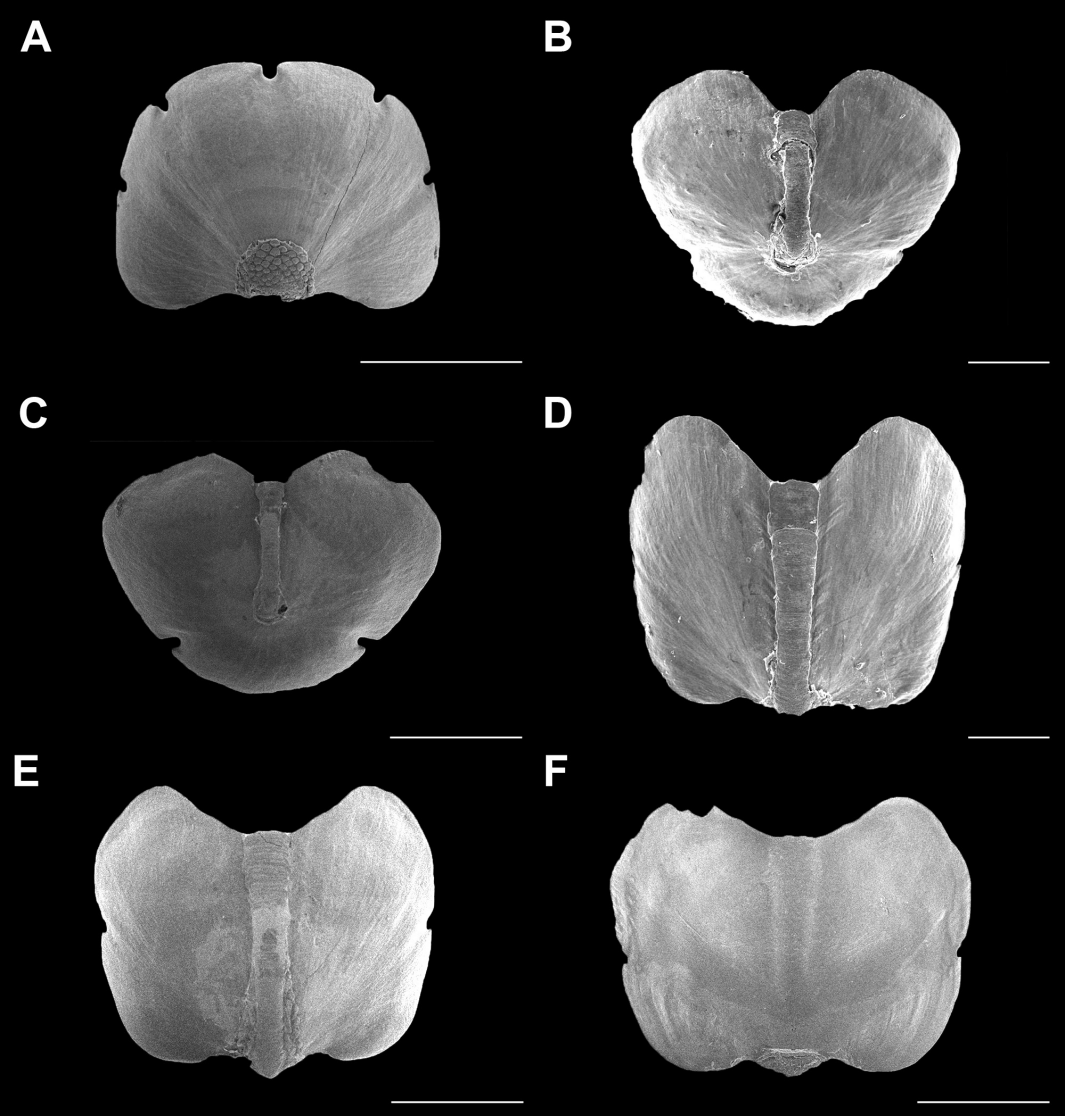
*Cryptoconchus floridanus* (Dall, 1889). Scanning electron images of a specimen from Isla Perez (CNMO4996, 9.3 mm length). **A** dorsal view of head valve (I), SB = 500 μm **C** dorsal view of tail valve (VIII), SB = 500 μm **E** dorsal view of intermediate valve IV, SB = 100 μm **F** ventral view of intermediate valve IV. Juvenile specimen from Isla Perez (CNMO4996, 7.1 mm length) **B** dorsal view of tail valve (VIII), SB = 100 μm **D** dorsal view of intermediate valve IV, SB = 100 μm.

**Figure 13.**
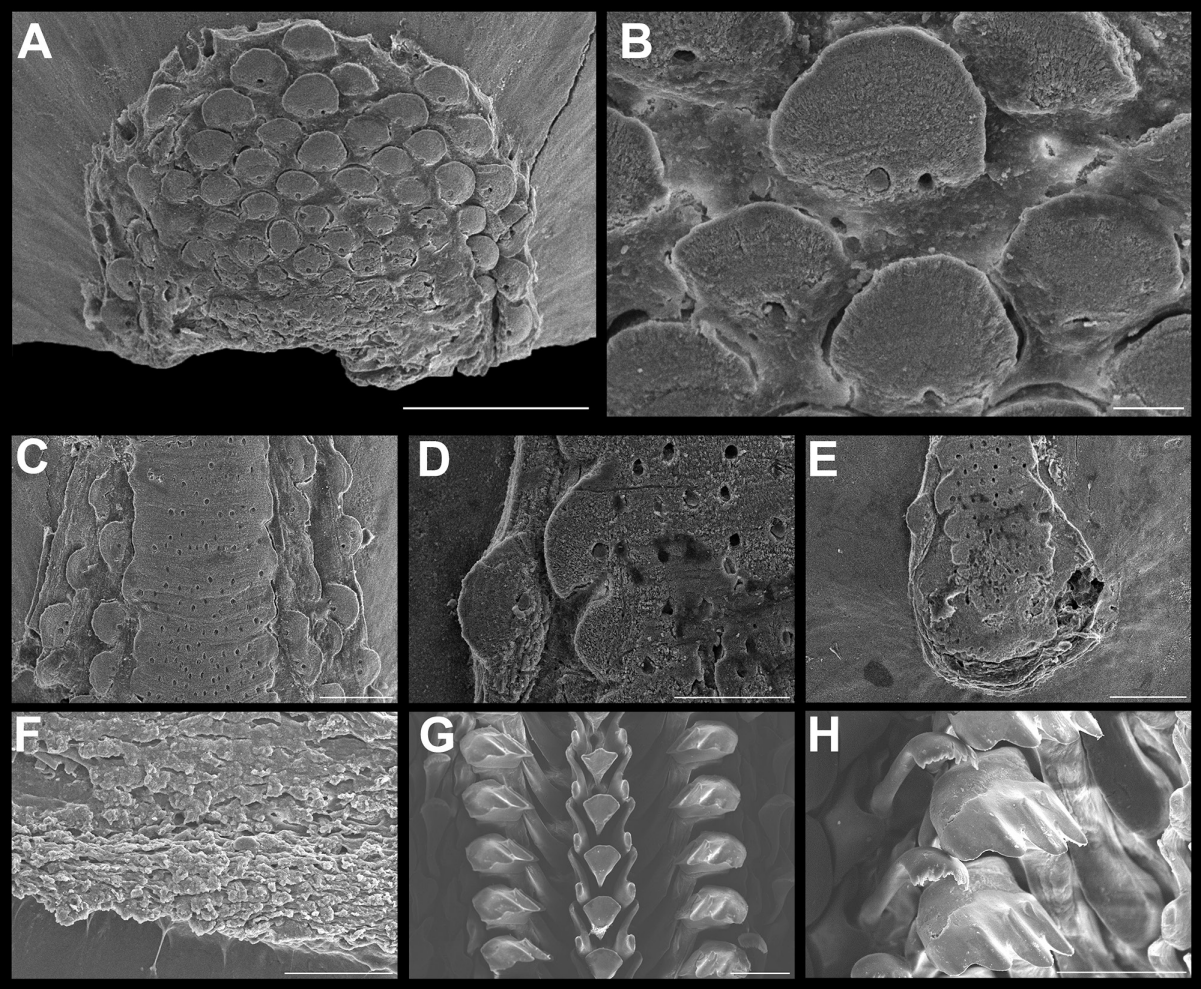
*Cryptoconchus floridanus* (Dall, 1889). Scanning electron images of specimen from Isla Perez (CNMO4996, 9.3 mm length). **A** tegmentum detail, showing the pustules on the head valve (I), SB = 200 μm **B** head valve pustules detail, SB = 200 μm **C** jugal area of intermediate valve IV, showing the pustule formation, SB = 200 μm **D** detail of pustules on jugal area of intermediate valve IV, SB = 200 μm **E** tail valve jugum of tail valve (VIII), SB = 200 μm **F** girdle detail, SB = 100 μm **G** radular teeth, SB = 100 μm **H** major lateral teeth detail, SB = 50 μm.

Figures 6L–O , 12A–F , 13A–H

######### Material examined.

Two specimens; 7.1–9.3 mm long, 3.0–4.2 mm wide. Isla Perez (CNMO4996).

######### Description.

Small-sized chitons, of oval body shape, somewhat elongated. Color dark brown to black, tegmentum white or creamy (Figure 6L–O). Valves covered by a smooth girdle; tegmentum very reduced; only the jugal area is exposed. Occasionally, tegmentum developing pustule formations, roundish or in irregular polygon shapes, opposed to oval, with a single megaesthete located on the pustule base. Head valve (Figure 12A) with a sub-quadrate outline, not notched, the posterior margin slightly concave; tegmentum semicircular and raised, flattened on its base; usually smooth, it can show small, irregularly shaped semi-oval pustules, which are larger anteriorly (Figure 13B), and arranged in a concentric pattern around the apex, covering most of the dorsal tegmentum surface (Figure 13A). Intermediate valves (Figure 12D, E), with a pointed apex; jugum smooth, slightly wider anteriorly; tegmentum when present reduced to two longitudinal narrow areas, adjacent to the jugum, and located near to the apex (Figure 13C), with small, longitudinally orientated and often irregularly rounded protruding (Figure 13D); jugum smooth with numerous megalaesthetes, distributed on its posterior end. Tail valve wider than long; jugal area narrow; mucro postmedian (Figure 12C); tegmentum (when present) bulb-shaped (Figure 13E); in juvenile specimens, the postmucronal area somewhat depressed, and slits missing (Figure 12B). Tegmentum around the mucro very limited, can bear a few pustule-like somewhat rounded to completely irregular shaped forms; the jugal area near to the mucro shows numerous megalaesthetes with no apparent arrangement (Figure 13E). Articulamentum wide, and especially in head valve, slits are somewhat deeply “u”-shaped; intermediate valves with two short and shallow slits (Figure 12F), almost absent in juveniles (Figure 12D), apophyses wide and wing-shaped; articulamentum of tail valve anteriorly wide, with short apophyses, with two “u”-shaped slits, located on the base of the valve; slit formula 5/1/2. Girdle smooth, constituted of mantle tissue, no elements present (Figure 13F). Radula (Figure 13G) with a central semi-wedge shaped tooth, rounded on its apical end and pointed posteriorly; major lateral tooth with four cusps, the outermost cusp only half of the size of the others, wider and broadened anteriorly (Figure 13H).

######### Habitat.

Found in the shallow subtidal to 12 m on rocks and dead coral, associated with crustose coralline red and green algae.

######### Remarks.

The reduced tegmentum area and its black nude girdle make the identification of this species quite unequivocal. The examination of the morphology of a juvenile (7.1 × 3.0 mm) PNAA specimen revealed the lack of slits and tegmentum pustules. In a somewhat larger animal (9.3 × 4.2 mm), the slits and pustules could be observed in all valves (explained above). Remarkably, the pustules on the head valve cover most of its area, whereas on the intermediate and the tail valve they are less numerous and more irregular in shape than on the head valve. Lyons (1988: figure 148, 149) figured some intermediate valves of a specimen of 10.7 mm length from Vaca Key, Monroe County, Florida with rudimentary pustules near the jugal area, which strongly resemble PNAA specimens. On the contrary, this condition was not observed in Puerto Rico specimens (García-Ríos 2003).

The examination of a larger *C. floridanus* (CNMO5560, 20. 3 × 8.3 mm) (Figure 6O) from Banco Chinchorro revealed a lack of pustules and little tegmentum development at all. Our observations suggest that some chitons might develop tegmentum, including pustules, but this in the observed specimens was not be related to chiton size. The Banco Chinchorro specimen of *C. floridanus* is one of the largest animals recorded from Mexico, after the Puerto Rico species of 21 mm length (García-Ríos 2003: figure 153), which also lacks of tegmentum formations. Regarding the radula, the fourth cusp of the major lateral tooth seems to be distinctive for the PNAA specimens, while the Puerto Rico specimen had only three cusps. The differences observed in our specimens compared to those from Puerto Rico, and the similarities with the PNAA and Florida Keys specimens is interpreted here to be due to high variability within a widespread species.

**Figure 14.**
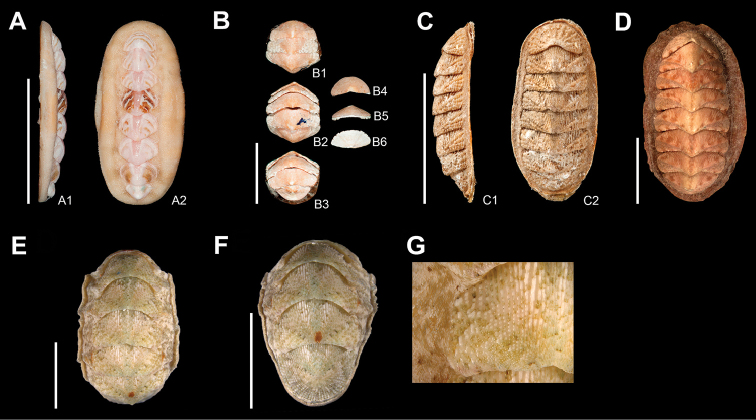
*Acanthochitona zebra* Lyons, 1988, **A** holotype (USNM859319), from Silver Cove Canal Freeport, Grand Bahama Island, specimen of 15 mm length; A1, in lateral view; A2, in dorsal view, SB = 10 mm. *Lepidochitona liozonis* (Dall & Simpson, 1901) **B** holotype (USNM161920), from Culebra Island, Ensenada Honda, Puerto Rico, specimen of 13.5 mm length; B1, curled chiton in dorsal view showing intermediate valves II-IV; B2, curled chiton in dorsal view showing intermediate valves IV-V; B3, curled chiton in dorsal view showing intermediate valves VI-VIII. Disarticulated valves; B4, head valve in dorsal view; B5, head valve in frontal view; B6, head valve in ventral view, SB = 5 mm. *Calloplax janeirensis* (Gray, 1828) **C** lectotype (NHMUK 1977041/2), from Brazil Rio de Janeiro, specimen of 16.2 mm length. C1, in lateral view and C2, in dorsal view, SB = 15 mm. *Tonicia schrammi* (Shuttleworth, 1856) **D** dorsal view of a paralectotype (NMBE19115/5a), from West Indies, Guadeloupe, specimen of 29 mm length, SB = 10 mm. *Stenoplax floridana* (Pilsbry, 1892), from Banco Chinchorro, Quintana Roo (CNMO5557) **E** specimen of 26.7 mm length, in dorsal view showing intermediate valves III-VII, SB = 5 mm **F** same specimen in dorsal view, showing intermediate valves VI-VIII, SB = 5 mm **G** detail of intermediate valve IV in dorsal view, showing the pustule formation near the diagonal ridge.

## Discussion

This study reports nine chiton species, belonging to five families and seven genera, in which Acanthochitonidae is the best represented with four species. PNAA chitons are characteristic for carbonate-rich habitats (Lyons and Moretzsohn 2009) and mainly associated with a rocky substrate in subtidal depths, with the exception of *I. erythronotus* found on dead coral. *Tonicia schrammi*, *C. janeirensis* and *L. liozonis* were exclusively reported from the intertidal, and *Acanthochitona* species showed more affinity to subtidal depths. We suspect that the low abundances recorded for *L. liozonis*, *T. schrammi* and *C. floridanus* and *A. roseojugum* in the original surveys, which mainly focused on other invertebrate groups, and all chiton material included in this paper was collected as bycatch.

Our research from PNAA produced *Tonicia schrammi* as the only representative of Chitonidae, and extends its distribution to the Gulf of Mexico. Other members of this family, specifically members of the genus *Chiton*, have been reported as widespread in the Caribbean (Bullock 1988) but were absent from our samples. This is probably due to the predominance of coral substrates and low rock formations with low tide activity in the PNNA habitats, whereas most species of *Chiton* are common in the intertidal of rocky shores.

A high morphological variability could be observed in the valve sculpture of *C. janeirensis* and *C. floridanus*, and in the central and minor lateral teeth in the radula of *T. schrammi*, *A. hemphilli*, and *C. floridanus*, which also show a higher similarity with specimens from the Florida Keys than with specimens from Puerto Rico and the Bahamas. Morphological differences were also identified within the same species from other regions within the Caribbean. We found a great accordance between *A. andersoni* and *A. roseojugum* descriptions, with very few valve and pustule character states that allow to separate both species. Further studies including a DNA analysis are needed to clarify the taxonomic status of these two taxa.

*Acanthochitona hemphilli* showed the most striking variation when comparing it to specimens from other areas, particularly in the repetitive lack of the second slit on tail valve with no apparent damage. Although it is known that members of Acanthochitonidae follow the general trend of tegmentum reduction and the extension of the articulamentum laminae, the loss of slits in the sutural laminae of *A. hemphilli* seems to represent a natural pattern within *Acanthochitona* s.s.

A study of the morphological variability within species of the Acanthochitonidae can be performed by comparison with García-Ríos (2003), who fully reviewed the species presented here and figured mostly specimens from Puerto Rico. *Cryptoconchus floridanus* from the Florida Keys (Saito 2004: figure 7A–F) had similar radular teeth morphology to specimens from Puerto Rico, with a sub-spatulate, distally wider central tooth and a tricuspid major lateral tooth. Instead, PNAA specimens revealed wedge-shaped central tooth and the major lateral with a fourth smaller outer cusp; which requires further investigation and the examination of more specimens.

Lyons (1988) described *Acanthochitona* species from the Caribbean and from few areas of Central America, focused exclusively on pustule morphology and aesthete number and position. Later, Watters (1990) also focused on the same pustule elements in the Eastern Pacific *Acanthochitona* species. Finally, García-Ríos (2003) included the radula and girdle elements to characterize Puerto Rican chitons, supplying a large compilation of SEM images that allow a much better understanding of the taxonomic structures of Caribbean species. However, in the major contributions dealing with *Acanthochitona* there was a tendency to omit other morphological characters and to limit the detailed description of a species exclusively to tegmentum sculpturing. This is the case of *A. zebra*, whose valves structure (dorsal and ventral) were described in detail (Lyons 1988), but with few other details of its morphology. We figured *A. zebra* girdle spicules and radular teeth for the first time, which are somewhat oval long and strongly ribbed, with few hyaline spicules in the girdle tufts, and the central tooth is sub-rectangular and the major lateral tooth is tricuspid.

The comparison of PNAA chiton diversity with that of other areas was challenging due to the limited number of specimens available for the study, and the lack of good images of morphological structures in the existing literature. We have included SEM images of radular and girdle features described here for the first time, especially for *Acanthochitona* species.

Saito (2004) also stressed the lack of radula information in most of the chiton studies, and he proposed a more refined study of the radular teeth. However, our examination of the central radular teeth revealed consistently high intraspecific variability for species of *Acanthochitona*. Still, we agree with Saito’s suggestion that it is desirable to generate a full set of information on the character states of the radula, as well as the girdle elements to increase the effectiveness of comparison within and between species; and we also coincide with other authors that more specimens are needed to be examined from different localities to verify or falsify previous species records.

Despite a long history of taxonomic mollusk research in the Caribbean, the chiton fauna of Mexican shores in the Yucatan Peninsula and its surroundings remains far from well known. We predict that future studies will extend the distribution range of other Caribbean species to include the PNAA reefs or their vicinity in the Gulf of Mexico.

## Museums’ collections

We are grateful for the prompt assistance on photograph the type specimens to: W. Moser curator of the Invertebrate Zoology collection of the United States National Museum of Natural History Smithsonian Institution, Washington D.C. and the technical assistance provided by Y. Villiacampa. To Ms. A. Salvador, curator of marine Mollusca at the British Museum of Natural History of London and to K. Webb from the NHMUK Photographic Unit.

## Supplementary Material

XML Treatment for Ischnochiton erythronotus

XML Treatment for Stenoplax bahamensis

XML Treatment for Calloplax janeirensis

XML Treatment for Tonicia schrammi

XML Treatment for Lepidochitona liozonis

XML Treatment for Acanthochitona hemphilli

XML Treatment for Acanthochitona roseojugum

XML Treatment for Acanthochitona zebra

XML Treatment for Cryptoconchus floridanus

## Appendix

Summary of chiton species occurring within the Atlantic coast of Mexico, the species distribution ranges are indicated north to south, first continental localities followed by Islands.

**Class Polyplacophora Gray, 1821**

**Order Chitonida Thiele, 1910**

**Suborder Chitonina Thiele, 1910**

**Family Ischnochitonidae Dall, 1889**

***Ischnochiton striolatus* (Gray, 1828)**

**Type locality.** Brazil, Río de Janeiro; between Bogue Inlet, North Carolina and Santa Catarina Islands (Kaas and Van Belle 1990).

**Additional localities.** USA (Florida Keys), Mexico (Yucatan, Quintana Roo); Cuba, Jamaica, Puerto Rico, St. John, St. Croix, Antigua, Guadeloupe, Dominica, Martinique, Grenada, Tobago, Bonaire, Klein Bonaire, Aruba, Colombia, Trinidad (Kaas 1972, Ferreira 1985, Reyes-Gómez and Salcedo-Vargas 2002, García-Ríos 2003, Lyons and Moretzsohn 2009).

***Ischnochiton erythronotus* (C. B. Adams, 1845)**

**Type locality.** Jamaica (Kaas and Van Belle 1990).

**Additional localities.** USA (Bonefish Key and Garden Key, Florida), Mexico (**PNAA**, Quintana Roo: Cozumel), Belize, Honduras; Bahamas, Cuba, Jamaica, Puerto Rico, St. Thomas, St. Eustatius, Guadeloupe, Barbados, Dominican Republic, Virgin Islands, Cayman Islands (Kaas 1972, Ferreira 1978a as *Chiton erythronotus*, 1985, Kaas and Van Belle 1990, Reyes-Gómez and Salcedo-Vargas 2002, García-Ríos 2003, Lyons and Moretzsohn 2009, present study).

***Ischnochiton pseudovirgatus* Kaas, 1972**

**Type locality.** Boca Lagoon, Curasao (Kaas and Van Belle 1990).

**Additional localities.** USA (Florida: Jupiter and St. Lucie); Mexico (Yucatan); Curacao, Trinidad, Barbados (Ferreira 1985, Kaas and Van Belle 1990, Lyons and Moretzsohn 2009).

***Stenoplax floridana* (Pilsbry, 1892)**

**Type locality.** Key West, Florida (Kaas and Van Belle 1987).

**Additional localities.** USA (Florida Keys), Mexico (Yucatan), Honduras, Panama, Colombia; Cuba, West Caribbean (Kaas & Van Belle, 1987, Lyons and Moretzsohn 2009).

***Stenoplax bahamensis* Kaas & Van Belle, 1987**

**Type locality.** Arthur´s Town, Cat Island, Bahamas (Kaas and Van Belle 1987).

**Additional localities.** USA (Florida Keys), Mexico (Yucatan, **PNAA**, Quintana Roo), Honduras; Bahamas, Cuba, Jamaica, Hispaniola, Isla de San Andres (as *S. producta* in Bullock 1985, Kaas and Van Belle 1987, Lyons and Moretzsohn 2009, present study).

***Stenoplax boogii* (Haddon, 1886)**

**Type locality.** West of Isla Plata, Colombia (Kaas and Van Belle 1987).

**Additional localities.** Western Atlantic: USA (Florida), Belize, Colombia, Brazil (Los Testigos, Fernando de Noronha and Ceara to Alagoas); Bermudas, Bahamas, Puerto Rico, Cayman Islands, Virgin Islands, Aruba. Eastern Pacific: Mexico (Cabo San Lucas), Colombia, Ecuador, Peru, Costa Rica, Panama (Kaas 1972, Ferreira 1985, Kaas and Van Belle 1987, Reyes-Gómez and Salcedo-Vargas 2002, García-Ríos 2003, Lyons and Moretzsohn 2009).

**Family Callistoplacidae Pilsbry, 1893**

***Calloplax janeirensis* (Gray, 1828)**

**Type locality.** Brazil Rio de Janeiro (Kaas and Van Belle 1994).

**Additional localities.** USA (Florida), Mexico (Yucatan, **PNAA**, Quintana Roo: Cozumel), Venezuela (Puerto Mara, Quetepec, Guiria, Carupano, Santa Fe, Coche Island and Margarita Island), Brazil (Porto Van Belle); Bahamas, Puerto Rico, Virgin Islands (San Martin, Saba, Dominica, Trinidad) (Ferreira 1978b, Kaas and Van Belle 1994, Bullock and Franz 1994, Bullock et al. 1994, Reyes-Gómez and Salcedo-Vargas 2002, García-Ríos 2003, as *Chaetopleura janeirensis* in Lyons and Moretzsohn 2009, present study).

**Family Chitonidae Rafinesque, 1815**

***Chiton tuberculatus* Linnaeus, 1758**

**Type locality.** Gambier, New Providence, Bahamas Island (Bullock 1988).

**Additional localities.** USA (Florida: Boca Raton), Mexico (Yucatan, Quintana Roo: Isla Mujeres), Venezuela (Isla de Margarita); Bermuda, Puerto Rico, Trinidad (Ferreira 1985, Bullock 1988, Reyes-Gómez and Salcedo-Vargas 2002, García-Ríos 2003, Lyons and Moretzsohn 2009).

***Chiton squamosus* Linnaeus, 1764**

**Type locality.** Robins Bay, St. Mary, Jamaica (Bullock 1988).

**Additional localities.** Mexico (Veracruz, Yucatan, Quintana Roo); Bahamas, Cuba, Jamaica (St. Mary Robin Bay), Grenada; (Bullock 1988, Reyes-Gómez and Salcedo-Vargas 2002, García-Ríos 2003, Lyons and Moretzsohn 2009).

***Chiton marmoratus* Gmelin, 1791**

**Type locality.** Unknown (Bullock 1988).

**Additional localities.** Mexico (Veracruz, Yucatan, Quintana Roo: Isla Mujeres), Panama; Costa Rica, Honduras, Venezuela; Bahamas, Cuba, Jamaica, Puerto Rico, Dominican Republic, Barbados, Guadeloupe, Grand Cayman, St. Thomas, St. Croix, Curacao, St. John, Saba, Antigua, St. Eustatius, Montserrat, Granada, Trinidad, Tobago, Bonaire (Klein Bonaire), Aruba (Kaas 1972, Ferreira 1985, Bullock 1988, Reyes-Gómez and Salcedo-Vargas 2002, García-Ríos 2003, Lyons and Moretzsohn 2009).

***Chiton viridis* Spengler, 1797**

**Type locality.** St. Croix, Virgin Islands (Bullock 1988).

**Additional localities.** USA (Florida Keys), Mexico (Yucatan, Quintana Roo), Panama, Colombia, Venezuela; Bahamas, Cuba, Puerto Rico, Barbados, St. Thomas, St. Croix, Grand Cayman, Curacao, Aruba Dominican Republic, Antigua St. Lucia, San Andres Island, Trinidad, Tobago Bonaire (Kaas 1972, Ferreira 1985, Bullock 1988, Reyes-Gómez and Salcedo-Vargas 2002, García-Ríos 2003, Lyons and Moretzsohn 2009).

***Acanthopleura granulata* (Gmelin, 1791)**

**Type locality.** Western Atlantic (Ferreira 1985).

**Additional localities.** USA (Florida), Mexico (Quintana Roo: Cozumel), Honduras, Nicaragua, Costa Rica, Panama; Bahamas, Cuba, Jamaica, Puerto Rico, Haiti, St. Martin, Curacao, St. John, St. Croix, St. Barts, St. Eustatius, St. Kitts, Antigua, Guadeloupe, Granada, Tobago, Grand Cayman, Trinidad, Dominican Republic, Trinidad, Bonaire, Dominican Republic (Kaas 1972, Ferreira 1985, Reyes-Gómez and Salcedo-Vargas 2002, García-Ríos 2003).

***Tonicia schrammi* (Shuttleworth, 1856)**

**Type locality.** Guadeloupe Island (Kaas et al. 2006).

**Additional localities.** USA (Florida), Mexico (**PNAA**), Honduras, Colombia; Bermuda, Bahamas, Cuba, Jamaica, Puerto Rico, Grand Cayman, Virgin Islands, Bonaire, Barbados, Aruba, Curacao, Guadalupe Island (Kaas 1972, Ferreira 1985, García-Ríos 2003, Kaas et al. 2006, Lyons and Moretzsohn 2009, present study).

**Family Lepidochitonidae Iredale, 1914**

***Lepidochitona liozonis* (Dall & Simpson, 1901)**

**Type locality.** Ensenada Honda, Isla Culebra, Puerto Rico (Kaas and Van Belle 1985).

**Additional localities.** USA (Florida), Mexico (Yucatan, **PNAA**, Quintana Roo), Colombia; Bermuda, Bahamas, Cuba, Puerto Rico, Barbados (Kaas and Van Belle 1985, García-Ríos 2003, Lyons and Moretzsohn 2009, present study).

**Family Mopaliidae Dall, 1889**

***Ceratozona squalida* (C. B. Adams, 1845)**

**Type locality.** Jamaica (Kaas and Van Belle 1994).

**Additional localities.** USA (Florida), Mexico (Quintana Roo: Cozumel), Colombia, Venezuela; Cuba, Bahamas, Jamaica, Puerto Rico, Barbados, Martinique, Grand Cayman Island, St. Vincent, St. John, Saba, St. Eustatius, Montserrat, Grenada, Trinidad, Tobago, Curacao, Aruba, Trinidad, Dominican Republic (Kaas 1972, Ferreira 1978a, 1985, Kaas and Van Belle 1994, Reyes-Gómez and Salcedo-Vargas 2002, García-Ríos 2003, Lyons and Moretzsohn 2009).

**Family Acanthochitonidae Pilsbry, 1893**

***Acanthochitona hemphilli* (Pilsbry, 1893)**

**Type locality.** Key West, Florida (Lyons 1988).

**Additional localities.** USA (Florida), Mexico (Yucatan, **PNAA**, Quintana Roo), Belize, Honduras; Bahamas, Cuba Puerto Rico; Jamaica, Aruba (Lyons 1988, Reyes-Gómez and Salcedo-Vargas 2002, García-Ríos 2003, Lyons and Moretzsohn 2009, present study).

***Acanthochitona pygmaea* Pilsbry, 1893**

**Type locality.** Cedar Keys, Florida (Lyons 1988).

**Additional localities.** USA (Florida: Cedar Keys), Mexico (Campeche, Yucatan, Quintana Roo); Bermuda, Bahamas, Cuba, Puerto Rico, Virgin Islands, Saba (Lyons 1988, García-Ríos 2003, Lyons and Moretzsohn 2009).

***Acanthochitona roseojugum* Lyons, 1988**

**Type locality.** Bartlett Hill, Eight Mile Rock, Grand Bahama Island (Lyons 1988)

**Additional localities.** USA (Florida, Dry Tortugas), Mexico (**PNAA**, Quintana Roo), Honduras; Bahamas, Cuba (Lyons 1988, García-Ríos 2003, Lyons and Moretzsohn 2009, present study).

***Acanthochitona zebra* Lyons, 1988**

**Type locality.** Silver Cove Canal, Freeport, Grand Bahama Island (Lyons 1988).

**Additional localities.** USA (Florida Keys, Dry Tortugas), Mexico (Yucatan, **PNAA**, Quintana Roo), Belize; Bahamas, Cuba Puerto Rico, Aruba, Curacao (Lyons 1988, García-Ríos 2003, Reyes-Gómez 2004, Lyons and Moretzsohn 2009, present study).

***Acanthochitona andersoni* Watters, 1981**

**Type locality.** St. Vincent, Lesser Antilles (Bullock 1981).

**Additional localities.** USA (Florida), Mexico (Quintana Roo, Yucatan), Panama, Venezuela; Bahamas, Cuba, Netherlands Antilles, (as *Americhiton andersoni* in Watters 1981, Lyons 1988, Reyes-Gómez and Salcedo-Vargas 2002, García-Ríos 2003, as *Americhiton andersoni* in Lyons and Moretzsohn 2009).

***Cryptoconchus floridanus* (Dall, 1889)**

**Type locality.** Cape, Florida (Lyons 1988).

**Additional localities.** USA (Florida Keys, Dry Tortugas), Mexico (Yucatan, **PNAA**); Bahamas, Cuba, Jamaica, Puerto Rico, Cayman Islands, Aruba, Bonaire (Lyons 1988, García-Ríos 2003, Reyes-Gómez 2004, Lyons and Moretzsohn 2009, present study).
